# Biogenic Silver Nanoparticles Strategically Combined With *Origanum vulgare* Derivatives: Antibacterial Mechanism of Action and Effect on Multidrug-Resistant Strains

**DOI:** 10.3389/fmicb.2022.842600

**Published:** 2022-05-06

**Authors:** Sara Scandorieiro, Bianca C. D. Rodrigues, Erick K. Nishio, Luciano A. Panagio, Admilton G. de Oliveira, Nelson Durán, Gerson Nakazato, Renata K. T. Kobayashi

**Affiliations:** ^1^Laboratory of Basic and Applied Bacteriology, Department of Microbiology, Center of Biological Sciences, Universidade Estadual de Londrina, Londrina, Brazil; ^2^Laboratory of Medical Mycology and Oral Mycology, Department of Microbiology, Center of Biological Sciences, Universidade Estadual de Londrina, Londrina, Brazil; ^3^Laboratory of Microbial Biotechnology, Department of Microbiology – Laboratory of Electron Microscopy and Microanalysis, Center of Biological Sciences, Universidade Estadual de Londrina, Londrina, Brazil; ^4^Institute of Biology, Universidade Estadual de Campinas, Campinas, Brazil

**Keywords:** oregano oil, carvacrol, thymol, *Fusarium oxysporum*, green nanotechnology, ESKAPEE pathogens, carbapenem resistance, MRSA

## Abstract

Multidrug-resistant bacteria have become a public health problem worldwide, reducing treatment options against several pathogens. If we do not act against this problem, it is estimated that by 2050 superbugs will kill more people than the current COVID-19 pandemic. Among solutions to combat antibacterial resistance, there is increasing demand for new antimicrobials. The antibacterial activity of binary combinations containing bioAgNP (biogenically synthesized silver nanoparticles using *Fusarium oxysporum*), oregano essential oil (OEO), carvacrol (Car), and thymol (Thy) was evaluated: OEO plus bioAgNP, Car plus bioAgNP, Thy plus bioAgNP, and Car plus Thy. This study shows that the mechanism of action of Thy, bioAgNP, and Thy plus bioAgNP involves damaging the membrane and cell wall (surface blebbing and disruption seen with an electron microscope), causing cytoplasmic molecule leakage (ATP, DNA, RNA, and total proteins) and oxidative stress by enhancing intracellular reactive oxygen species and lipid peroxidation; a similar mechanism happens for OEO and Car, except for oxidative stress. The combination containing bioAgNP and oregano derivatives, especially thymol, shows strategic antibacterial mechanism; thymol disturbs the selective permeability of the cell membrane and consequently facilitates access of the nanoparticles to bacterial cytoplasm. BioAgNP-treated *Escherichia coli* developed resistance to nanosilver after 12 days of daily exposition. The combination of Thy and bioAgNP prevented the emergence of resistance to both antimicrobials; therefore, mixture of antimicrobials is a strategy to extend their life. For antimicrobials alone, minimal bactericidal concentration ranges were 0.3–2.38 mg/ml (OEO), 0.31–1.22 mg/ml (Car), 0.25–1 mg/ml (Thy), and 15.75–31.5 μg/ml (bioAgNP). The time-kill assays showed that the oregano derivatives acted very fast (at least 10 s), while the bioAgNP took at least 30 min to kill Gram-negative bacteria and 7 h to kill methicillin-resistant *Staphylococcus aureus* (MRSA). All the combinations resulted in additive antibacterial effect, reducing significantly minimal inhibitory concentration and acting faster than the bioAgNP alone; they also showed no cytotoxicity. This study describes for the first time the effect of Car and Thy combined with bioAgNP (produced with *F. oxysporum* components) against bacteria for which efficient antimicrobials are urgently needed, such as carbapenem-resistant strains (*E. coli*, *Klebsiella pneumoniae*, *Acinetobacter baumannii*, and *Pseudomonas aeruginosa*) and MRSA.

## Introduction

Multidrug-resistant bacteria have become a global clinical and public health problem. There are several possible causes of antibacterial resistance such as natural selection, mutations, antibiotic selection pressure due to overuse, fast spread of resistance by bacterial horizontal gene transfer and rapid reproduction, patients not finishing entire antibiotic course, absence of new antimicrobials being discovered, and others (O’Neill, 2016; Centers for Disease Control and Prevention [CDC], 2020a; World Health Organization [WHO], 2020). Infections due to multidrug-resistant pathogens prolong the length of hospital stay, cause hospital outbreaks, increase economic costs for healthcare, and cause high morbidity and mortality (Peters et al., 2019; Centers for Disease Control and Prevention [CDC], 2020b; World Health Organization [WHO], 2020). If no action is taken, it is estimated that by 2050 one person will die every 3 s, causing 10 million deaths a year (O’Neill, 2016). Multidrug-resistant bacteria, plus the lack of effectiveness of current antimicrobials in treating infections, are an emerging disaster to public health. The coronavirus disease-2019 (COVID-19) pandemic reinforces the need for research and development of new antimicrobials with potential to combat resistant bacteria and prevent emergence of their resistance, since respiratory viral infections may lead to secondary bacterial infections (Centers for Disease Control and Prevention [CDC], 2020b; Mirzaei et al., 2020).

The ESKAPEE (*Enterococcus faecium*, *Staphylococcus aureus*, *Klebsiella pneumoniae*, *Acinetobacter baumannii*, *Pseudomonas aeruginosa*, *Enterobacter* spp., and *Escherichia coli*) pathogens are commonly associated with antimicrobial resistance and are the leading cause of nosocomial infections worldwide (Partridge et al., 2018; Mulani et al., 2019). Multidrug-resistant strains have been found in domestic and industrial wastewater, rivers, lakes, and food for human consumption where they colonize, and they infect humans at community level. Therefore, it is not a problem restricted to hospitals; it also affects the environment where we live in (de Campos et al., 2018; Vivas et al., 2019; Centers for Disease Control and Prevention [CDC], 2020c; Rodríguez-Serrano et al., 2020). The CDC and the WHO have highlighted some ESKAPEE strains among microorganisms for which antimicrobials are urgently needed (Centers for Disease Control and Prevention [CDC], 2020b; World Health Organization [WHO], 2020).

In Gram-negative bacteria, enzymatic degradation by β-lactamases is a prevalent and efficient mechanism against antimicrobials, since enzymes remain and are concentrated in the periplasmic space of these bacteria (Ruppé et al., 2015; Codjoe and Donkor, 2018). Carbapenem-resistant strains have challenged healthcare settings; these microorganisms produce carbapenemases that extremely reduce treatment options, since they inactivate penicillins, cephalosporins, monobactams, and carbapenems, namely, imipenem, meropenem, doripenem, ertapenem, panipenem, and biapenem (Codjoe and Donkor, 2018; Nordmann and Poirel, 2019). Gram-positive strains also secrete β-lactamases; once these enzymes are not concentrated in bacterial cells, a common mechanism of resistance to β-lactam occurs because of alteration or replacement of an antibiotic target site (e.g., enzyme or cell wall). Methicillin-resistant *S. aureus* (MRSA) harbors the *mec*A gene, which encodes alternative transpeptidases with very low affinity for several β-lactams. These enzymes are encoded by mobile genetic elements that may carry resistance genes to other non-β-lactam antimicrobials (e.g., quinolones, tetracyclines, cotrimoxazole, trimethoprim, and aminoglycosides), reducing treatment options (Turlej et al., 2011). Bacteria easily exchange genes by horizontal gene transfer, spreading multidrug-resistance among different strains and genera (Turlej et al., 2011; Dhillon and Clark, 2012; Patel and Bonomo, 2013; Munita et al., 2015; Foster, 2017; Codjoe and Donkor, 2018; Nordmann and Poirel, 2019).

Antimicrobial alternatives to combat bacterial resistance include bioactive compounds from several natural sources (e.g., animals, plants, bacteria, and fungi), metallic nanoparticles, and antimicrobial combinations (Cardozo et al., 2013; Biasi-Garbin et al., 2015; Scandorieiro et al., 2016; Swamy et al., 2016; Pisoschi et al., 2017; Tyers and Wright, 2019; Raveau et al., 2020; Sánchez-López et al., 2020; Iseppi et al., 2021).

Combinations of antimicrobials are potential strategies to prevent the emergence of resistant strains and reduce unwanted characteristics of compounds (odor, taste, toxicity, or costs) (Fischbach, 2011; Bollenbach, 2015; Suzuki et al., 2017; Raymond, 2019; Tyers and Wright, 2019). In a previous study, our research group has reported that a combination containing *Origanum vulgare* (oregano) essential oil (OEO) and bioAgNP (silver nanoparticles biologically synthetized with *Fusarium oxysporum* components) show excellent activity against Gram-positive and Gram-negative bacteria, and has synergistic effect against extended-spectrum beta-lactamase (ESBL)-producing *E. coli* and carbapenemase (KPC)-producing *K. pneumoniae* (Scandorieiro et al., 2016).

OEO and its main bioactive components (carvacrol, Car; thymol, Thy) stand out among plant essential oils as excellent bactericidal agents against several strains including multidrug-resistant microorganisms (Nazzaro et al., 2013; Magi et al., 2015; Scandorieiro et al., 2016; Khan et al., 2017, 2019; Wijesundara and Rupasinghe, 2018; Sim et al., 2019; Cho et al., 2020; Xiao et al., 2020). Because of their strong antimicrobial property, oregano derivatives have a potential to be applied as antibiotics in the food industry (Alvarez et al., 2014; Gavahian et al., 2018; Karam et al., 2019), cosmetic products (Suzuki et al., 2015; Laothaweerungsawat et al., 2020), and human and veterinary clinical and hospital sectors (Mir et al., 2019; Tahmasebi et al., 2019; Arafa et al., 2020; Laothaweerungsawat et al., 2020). However, terpenoids derived from oregano present strong taste and smell, which may limit their use; therefore, strategies are required to minimize these undesirable organoleptic effects (Alvarez et al., 2014; Kotronia et al., 2017).

For centuries, humans have made use of metal antimicrobial properties. Silver containers were used by ancient civilizations (Persians, Phoenicians, Greeks, Romans, and Egyptians) to preserve food and water. Silver has also been used to treat eye infections and wounds (Alexander, 2009; Lemire et al., 2013). Nanotechnology has provided possibilities to revive the use of silver to combat microbial resistance. Silver nanoparticles, which have been used for over 100 years and are currently incorporated into various products for our daily lives, have been studied to combat several pathogens. Silver nanoparticles have large surface area and high oxidation-reduction potential, which provide them great effect against bacteria, including multidrug-resistant strains (Nowack et al., 2011; Cardozo et al., 2013; Biasi-Garbin et al., 2015; Ebrahiminezhad et al., 2016; Scandorieiro et al., 2016; Calderón-Jiménez et al., 2017; Sim et al., 2018; Simbine et al., 2019; Dalir et al., 2020). Because of their antimicrobial property, silver nanoparticles have been widely explored by the nanotechnology industry, since they, among other applications, are incorporated in the formulation of surface cleaners, textiles, toys, air and water disinfectants, antimicrobial inks, food packaging, wound dressing and other materials for cutaneous infections, coating for bone implants, dental prostheses, and catheters (Calderón-Jiménez et al., 2017; Nakazato et al., 2017, 2020; Kobayshi et al., 2019). Other physicochemical properties of silver nanoparticles (high electrical and thermal conductivity, catalytic activity, and non-linear optical properties) allow for the development of new products and scientific applications (Calderón-Jiménez et al., 2017).

Green nanotechnology has led to silver nanoparticle production with eco-friendly and low-cost methods (Durán et al., 2005; de Lima et al., 2012; Marcato et al., 2013; Behravan et al., 2019; Nisar et al., 2019). Our research group has shown that silver nanoparticles, synthesized with *F. oxysporum* components as reducing and capping agents (bioAgNP), have activity against bacteria, fungi, and protozoa, including multidrug-resistant strains (Cardozo et al., 2013; Biasi-Garbin et al., 2015; Longhi et al., 2016; Scandorieiro et al., 2016; Bocate et al., 2019; Machado et al., 2020). Despite their excellent antimicrobial activity, resistance to ionic silver and silver nanoparticles is already reported (Losasso et al., 2014; Graves et al., 2015; Panáček et al., 2017; Muller, 2018).

This evaluated, for the first time, the antibacterial effect of two double-drug combinations containing bioAgNP and oregano bioactive terpenoids (Car and Thy) against some ESKAPEE strains. A study on the mechanism of action against reference *E. coli* was performed for a combination composed of Thy and bioAgNP; its potential to prevent the emergence of resistance was also analyzed.

## Materials and Methods

### Bacterial Strains

Bacterial reference strains mainly from American Type Culture Collection (ATCC) and clinical isolates were used in this study. The reference strains were as follows: *E. coli* ATCC 25922, *K. pneumoniae* ATCC 10031, *P. aeruginosa* ATCC 9027, *A. baumannii* ATCC 19606, methicillin-sensitive *S. aureus* ATCC 25923, and MRSA N315. All clinical isolates tested were multidrug-resistant bacteria (KPC-producing *E. coli*, KPC-producing *K. pneumoniae*, carbapenem-resistant *P. aeruginosa*, and carbapenem-resistant *A. baumannii*); their antimicrobial susceptibility profile and origin are shown in Supplementary Table 1. All bacterial samples were stored in a Brain Heart Infusion (BHI, Acumedia) broth containing 25% (v/v) glycerol (Merck) at −80°C.

### Antimicrobial Agents

#### Oregano-Derived Compounds

*Oregano vulgare* (oregano) essential oil was obtained from Ferquima Industry and Commerce of Essential Oil (São Paulo, Brazil). Oil (batch 227) was extracted by steam distillation, and its main components (72% carvacrol, 2% thymol, 4.5% gamma-terpinene, 4% para-cymene, and 4% linalool) were described in a company technical report. Carvacrol-W224502 (Car) and thymol-T0501 (Thy) were purchased from Sigma-Aldrich. Both OEO and Car were in the liquid state. To express their concentrations in mass per volume (mg/ml), density was assumed to be 0.95 g/ml for OEO and 0.976 g/ml for Car based on the technical report. Thy was in powder form. Individual stock solutions of OEO at 50% (v/v), Car at 50% (v/v), and Thy at 40 mg/ml were prepared in dimethyl sulfoxide (DMSO, Sigma-Aldrich). DMSO maximum concentration in assays was 2.5% (v/v).

#### Biogenically Synthetized Silver Nanoparticles

Biologically synthetized silver nanoparticles (bioAgNP) were obtained according to a previously established method (Durán et al., 2005). *F. oxysporum* (strain 551), from ESALQ-USP Genetic and Molecular Biology Laboratory (Piracicaba, São Paulo, Brazil), was used to prepare bioAgNP. A fungus was grown in a medium containing 0.5% (w/v) yeast extract (Becton, Dickinson and Company), 2% (w/v) malt extract, 2% (w/v) agar (Acumedia), and distilled water at 28°C for 7 days. *F. oxysporum* biomass was added to distilled water at 0.1 g/ml and incubated at 28°C for 72 h in agitation (150 rpm). Thereafter, aqueous solution components were separated by vacuum filtration (qualitative filter having average pore size of 4 to 12 μm, Unifil). AgNO_3_ (Sigma-Aldrich) at 0.01 M was added to this solution. The system was kept at 28°C for 15 days under static conditions. BioAgNP were obtained after reduction of silver nitrate by fungal-free solution components. Aliquots of the system were removed for measuring absorption spectra using an ultraviolet-visible spectrophotometer (Thermo Scientific™ Multiskan™ GO Microplate Spectrophotometer) to verify the surface plasmon resonance peak of the bioAgNP. Washing of the bioAgNP was performed by centrifugation (27,000 × *g*, 4°C, 30 min) followed by incubation in ultrasonic bath (30 min); both steps were repeated three times. Ag quantification was performed with Energy Dispersive X-ray Fluorescence Spectrometer EDX-7000. Nanoparticle diameter was determined by photon correlation spectroscopy using ZetaSizer NanoZS (Malvern), and zeta potential measurement was performed using the same instrument. Scanning electron microscopy (SEM, FEI Quanta 200) was performed for bioAgNP morphology analysis.

### Minimum Inhibitory Concentration of Compounds Separately

Determination of the minimum inhibitory concentration (MIC) of each antimicrobial (OEO, Car, Thy, bioAgNP, and AgNO_3_) was performed with broth microdilution method according to Clinical and Laboratory Standards Intitute [CLSI] (2015) guidelines, with necessary modifications. Tested concentration ranges were as follows: (i) 0.07–9.5 mg/ml for OEO, (ii) 0.08–9.76 mg/ml for Car, (iii) 0.008–1 mg/ml for Thy, (iv) 0.49–63 μg/ml for the bioAgNP, and (v) 1.33–17 μg/ml for AgNO_3_. AgNO_3_ (Sigma-Aldrich) antibacterial activity was analyzed for comparison with the bioAgNP. Müeller-Hinton broth (MHB, Difco) alone and MHB containing each of the antimicrobials separately were tested as sterility controls. Untreated bacteria inoculated on the MHB alone and containing DMSO at 1.25% (v/v) were tested as growth control with solvent. MIC was defined as the lowest antimicrobial concentration that inhibited visible growth after 24 h of treatment at 37°C. All assays were performed in triplicate, at least on three different occasions against reference and multidrug-resistant bacterial strains.

### Minimum Bactericidal Concentration of Compounds Separately

Minimal bactericidal concentration (MBC) of each antimicrobial was determined by subculturing 10 μL from broth dilution MIC and above concentrations in Müeller-Hinton agar (Acumedia) without antimicrobials. MBC was defined as the lowest concentration required to kill ≥99.9% of bacteria after 24 h of antimicrobial treatment (National Committee for Clinical Laboratory Standards [NCCLS], 1999). All assays were performed in triplicate, at least on three different occasions against reference and multidrug-resistant bacterial strains.

### Antibacterial Combination Assay

Four antimicrobial combinations were tested and as follows: (i) OEO and bioAgNP, (ii) Car and bioAgNP, (iii) Thy and bioAgNP, and (iv) Car and Thy. The antibacterial interaction of both combined compounds was determined by broth dilution in double-antimicrobial gradient as described by Traub and Kleber (1975), with necessary modifications. To standardize inoculum density for susceptibility test, isolated bacterial colonies grown in the MHA medium were suspended in a saline solution (0.9% NaCl, w/v, Merck), and this suspension was adjusted to achieve turbidity equivalent to 0.5 McFarland standard, which corresponds approximately to 1.5 × 10^8^ colony-forming units (CFU)/ml. The equivalent 0.5 McFarland suspension was diluted 1:100 in MHB to obtain a concentration of approximately 10^6^ CFU/ml. A volume of 0.05 ml of bacterial inoculum at 10^6^ CFU/ml was added to 0.05 ml of the MHB containing antimicrobial combinations and whose final concentrations ranged as follows: (i) 0.02–1.19 mg/ml for OEO, (ii) 0.02–0.61 mg/ml for Car, (iii) 0.03–0.5 mg/ml for Thy, and 0.98–15.75 μM for the bioAgNP. Lastly bacteria at 5 × 10^5^ CFU/ml in the MHB with double antimicrobials in combination were incubated at 37°C for 24 h. Sterilization and growth control were performed as described above for MIC determination assay. The antibacterial interaction of both compounds in each combination was analyzed with fractional inhibitory concentration index (FICI) according to Yadav et al. (2013) using Equation 1:


$$\text{FICI}={\text{FIC}}_{1st\mathrm{antimicrobial}}+{\text{FIC}}_{2nd\mathrm{antimicrobial}}$$

$$FI\text{C}={\text{MIC}}_{\text{combination}}/{\text{MIC}}_{\text{individual}}$$

Equation 1. Formula for finding FICI value.

The interaction based on FICI is interpreted as follows: “synergistic” if FICI ≤ 0.5, “additive” if >0.5 and ≤1, “indifferent” (no interaction) if >1 and ≤2, and “antagonist” if >2. All assays were performed in triplicate, at least on three different occasions against reference and multidrug-resistant bacterial strains.

### Time-Kill Assay

Time-kill assay was performed using the viable cell count method and according to the (National Committee for Clinical Laboratory Standards [NCCLS], 1999). Five conditions of treatment were tested: bacterial cultures treated with individual antimicrobials (OEO, Car, Thy, and bioAgNP) and the combination of Thy and bioAgNP. Bacterial culture with no antimicrobial was tested as growth control. At nine time points (0 h, 10 s, 10 and 30 min, and 2, 4, 7, 10, and 24 h) of treatment at 37°C, 10 μl from serial dilutions (in the saline solution composed of 0.9% NaCl, w/v) of treated and non-treated cultures were subcultured in MHA for CFU/ml determination. Antimicrobials alone in MBC and combination of Thy and bioAgNP at 0.5 × MIC (additive interaction) were tested against bacterial strains with an initial cellular density of 5 × 10^5^ CFU/ml.

For *E. coli* ATCC 25922, antimicrobial tested concentrations were 0.3 mg/mL (OEO), 0.3 mg/mL (Car), 0.25 mg/mL (Thy), 15.75 μg/mL (bioAgNP), and combination of Thy at 0.12 mg/mL plus bioAgNP at 7.88 μg/mL. For carbapenemase-producing *K. pneumoniae* KPC 5795, tested concentrations were 0.59 (OEO), 0.61 (Car), 0.5 mg/ml (Thy), and 31.5 μg/ml (bioAgNP), and the combination of Thy and bioAgNP at 0.25 and 15.75 μg/ml, respectively. For carbapenem-resistant *A. baumannii*, tested concentrations were 1.19 (OEO), 1.22 (Car), and 0.25 mg/ml (Thy), and 15.75 μg/ml (bioAgNP). For MRSA N315, concentrations were 1.19 (OEO), 1.22 (Car), and 1 mg/ml (Thy), and 31.25 μg/ml (bioAgNP). All assays were carried out in triplicate, at least on two different occasions.

### Characterization of Antibacterial Action Mechanism

Characterization of antibacterial mode of action was performed on five treatments: (i) OEO at 0.15, (ii) Car at 0.15, and (iii) Thy at 0.12 mg/ml, (iv) and bioAgNP at 15.75 and (v) the combination containing Thy and bioAgNP at 0.23 and 7.88 μg/ml, respectively. Previously, time-kill assays have been carried out to determine subinhibitory antimicrobial concentrations that do not inhibit bacterial growth, as shown in Supplementary Table 2. The antimicrobials were tested against *E. coli* ATCC 25922 at approximately 10^9^ CFU/ml at 25°C. The treatments were tested in phosphate-buffered saline (0.1 M PBS, pH 7.2) in the time-kill and other characterization assays (see sections “Measurement of Reactive Oxygen Species,” “Evaluation of Lipid Peroxidation,” “Quantification of Extracellular ATP Levels,” “Quantification of Biomolecules Leakage,” “Cellular Alterations Seen by Electron Microscopy”). PBS was composed of 0.9% (w/v) NaCl, 0.2 M monobasic sodium phosphate (Chemco), and 0.2 M dibasic sodium phosphate (Nuclear). PBS alone (untreated bacterial) was used as control.

#### Measurement of Reactive Oxygen Species

Production of reactive oxygen species (ROS) by treated and untreated (control) bacteria was evaluated using fluorescent dye-based assay (total ROS Assay Kit 520 nm; Thermo Fisher Scientific, Carlsbad, CA, United States), according to the manufacturer’s recommendations. Briefly, 2 ml bacterial cells at approximately 10^9^ CFU/ml were pelleted (5,500 × *g*, 6 min, 25°C) and resuspended with an ROS stain solution at a final concentration of 1×. Thereafter, the cells were incubated to be labeled (37°C, 1 h, in absence of light). After label time, extracellular fluorescent dye was removed with two washing steps by centrifugation (5,500 × *g*, 6 min, 25°C) and PBS. Finally, the labeled cells were resuspended in 1 ml of PBS. A volume of 0.1 ml of the labeled cells was added to 0.1 ml of PBS containing the antimicrobials (treatments) or PBS alone (untreated control). At nine time points of treatment (0, 15, 30, 45, 60, 75, 90, 105, and 120 min), fluorescence emission was measured at 520 nm using a fluorescent microplate reader (PERKIN ELMER 1420 MULTILABEL COUNTER VICTOR 3) with an excitation wavelength of 495 nm. The experiment was conducted in triplicate, at least on three different occasions.

#### Evaluation of Lipid Peroxidation

Lipid peroxidation was evaluated by malondialdehyde (MDA) quantification [Lipid Peroxidation (MDA) Assay Kit; Sigma-Aldrich, St. Louis, MO, United States], according to the manufacturer’s recommendations. Briefly, bacteria at 10^9^ CFU/ml were pelleted (5,500 × *g*, 6 min, 25°C) and resuspended in PBS containing the antimicrobials or alone. After three time points of treatment (1, 2, and 3 h), bacterial cells of each sample were pelleted and resuspended in MDA lysis buffer containing 1× butylated hydroxytoluene (BHT). The insoluble material was removed by centrifugation (13,000 × *g*, 10 min), and a volume of 0.2 ml of the supernatant was added to 0.6 ml of a thiobarbituric acid (TBA) solution. Then, incubation at 95°C for 1 h was performed to form the MDA-TBA adduct. The samples were cooled to room temperature in an ice bath for 10 min; 0.2 ml of the reaction mixture was transferred into a 96 well plate, which was read at 532 nm (Thermo Scientific™ Multiskan™ GO Microplate Spectrophotometer). Known concentrations of MDA were used to construct a calibration curve. The concentration of MDA in each sample (treated and untreated) was determined by linear regression analysis. PBS was used as control. The assay was carried out in triplicate, at least on three different occasions.

#### Quantification of Extracellular ATP Levels

Extracellular ATP levels of *E. coli* ATCC 25922 (treated and untreated) were determined by luciferin-luciferase bioluminescence assay according to the manufacturer’s recommendations (ATP Determination Kit, A22066; Molecular Probes, Eugene, OR, United States). Briefly, *E. coli* at 10^9^ CFU/ml were pelleted (5,500 × *g*, 6 min, 25°C), and a new inoculum at 2 × 10^10^ CFU/ml (20× concentrated) was prepared in PBS (0.1 M pH 7.2). A volume of 0.05 ml of 20× concentrated bacteria was added to 0.05 ml of PBS containing the antimicrobials or PBS alone (control). At 0 h of treatment, a volume of 0.01 ml of each sample (treated or untreated bacteria) was mixed to 0.09 ml of a standard reaction solution (which contained reaction buffer at 1×, 1 mM DTT, 0.5 mM D-luciferin, 1.25 μg/ml luciferase, and water) in a 96-well black microplate. At five time points of treatment (at 25°C), fluorescence emission was measured at 560 nm using a fluorescent microplate reader (PERKIN ELMER 1420 MULTILABEL COUNTER VICTOR 3). Standard curves for ATP concentrations were constructed, and by linear regression analysis the concentration of ATP in each sample (treated and untreated) was determined. The assay was carried out in duplicate, at least in three different occasions.

#### Quantification of Biomolecules Leakage

Untreated and treated *E. coli* at 10^9^ CFU/ml were incubated for 30 min, then bacterial cells were pelleted (5,500 × *g*, 6 min, 25°C). The supernatant was analyzed by quantification of several extracellular biomolecules, total proteins, single-stranded DNA (ssDNA), double-stranded DNA (dsDNA), and RNA using Thermo Scientific™ NanoDrop Lite Spectrophotometer.

#### Cellular Alterations Seen by Electron Microscopy

For scanning electron microscopy (SEM) and transmission electron microscopy (TEM) analyses, sample preparation (treated and untreated bacteria) was performed according to de Oliveira et al. (2011). For the SEM study, *E. coli* was exposed to five different treatments for 30 min. After treatment time, 0.25 ml of bacterial cell suspensions of each sample were spotted onto poly-L-lysine (Sigma-Aldrich)-coated glass slides. Thereafter, each sample was fixed (for 20 h at 4°C) by immersion in 1 ml of 0.1 M sodium cacodylate buffer (pH 7.2) containing 2.5% (v/v) glutaraldehyde and 2% (v/v) paraformaldehyde, following post-fixation in OsO_4_ 1% for 2 h at room temperature. All reagents for both chemical fixations were provided by Electron Microscopy Sciences. After both fixation and post-fixation, three washing steps (15 min each) with a sodium cacodylate buffer were performed. Post-fixed cells were dehydrated in an ethanol gradient (Sigma-Aldrich) (30, 50, 70, 90, and 100°GL), critical point-dried using CO_2_ (BALTEC CPD 030 Critical Point Dryer), coated with gold (BALTEC SDC 050 Sputter Coater), and observed under a scanning electron microscope (FEI Quanta 200).

For the TEM study, *E. coli* ATCC 25922 at 10^9^ CFU/ml was exposed to the bioAgNP (7.88 μg/ml) for 1 h, and bacteria in PBS were used as untreated control. After treatment, cells from 1 ml were washed twice with PBS by centrifugation (5,500 × *g*, 4°C, 10 min), and fixed, post-fixed and dehydrated in an ethanol gradient as described for SEM. The samples were embedded and blocked in Araldite. The block was cut into ultrathin sections of 60–70 nm (Leica ULTRACUT UCT Leica UCT) that were stained with 2% uranyl acetate for 15 min, washed with distilled water, post-stained with 0.2% lead citrate for 15 min, washed with distilled water again, and observed under a Zeiss EM900 transmission electron microscope. At least 18 microscopic fields were observed for each sample.

### Cytotoxicity Assay

Hemolytic activity of the antimicrobials alone (OEO, Car, Thy, and bioAgNP) and in combinations (OEO plus bioAgNP, Car plus bioAgNP, Thy plus bioAgNP, and Car plus Thy) was determined according to Izumi et al. (2012), with necessary modifications. The assay was also performed for the *F. oxysporum*-free filtrate used in bioAgNP synthesis. Blood was obtained in tubes containing sodium heparin (Vacuplast) from healthy human donors with voluntary consent, which was approved by the human ethics committee (CAAE 47661115.0.0000.5231, No. 1.268.019 – UEL). Erythrocytes were separated by centrifugation (5,500 × *g*, 4°C, 5 min), and they were inoculated at 3% (v/v) in 96-well plates containing PBS (0.1 M, pH 7.2) with different antimicrobials whose final concentrations ranged as follows: 0.07–9.5 mg/ml for OEO, 0.08–9.76 mg/ml for Car, 0.005–1 mg/ml for Thy, 0.98–126 μg/ml for bioAgNP, 0.15–20% (v/v) for the *F. oxysporum*-free filtrate, and 2.66–340 μg/ml for AgNO_3_. After 3 h of incubation at 37°C, supernatants were read at 550 nm to evaluate the release of hemoglobin. Triton X-100 (Sigma-Aldrich) at 1% (v/v) was used as positive control for 100% hemolytic activity, and PBS with no antimicrobial was used as negative control. The hemolysis percentage of each antimicrobial treatment was calculated using Equation 2:


Hemolysis(%)=A/B×100

$$A=OD_{550}treatedsample-OD_{550}negativecontrol$$

$$B=OD_{550}positivecontrol-OD_{550}negativecontrol$$

Equation 2. Formula for finding hemolysis percentage.

Fifty percent cytotoxicity concentration (CC_50_) was defined as the antimicrobial concentration required to cause 50% of hemolysis compared to untreated control. CC_50_ of the individual antimicrobials was determined by regression analysis.

### Prolonged Exposure of Bacteria to Thymol and Biogenically Synthesized Silver Nanoparticles

Prolonged exposure of *E. coli* ATCC 25922 to Thy and the bioAgNP (individually and in combination) was carried out to analyze if the bacteria develop fast tolerance to antimicrobials. Before starting the experiment, *E. coli* was grown in MHB in a shaking incubator (130 rpm) at 37°C for 72 h (every 24 h, the medium was renewed). Then, *E. coli* was exposed for 25 days to the antimicrobials at 37°C and 130 rpm. Daily transfers of 0.05 ml of each previous culture into 0.95 ml of MHB alone (untreated control) or containing the antimicrobials were carried out. At the beginning of experiment, bacterial samples were exposed to subinhibitory concentrations of the antimicrobials, then the concentrations were increased gradually. All details of daily antimicrobial concentrations are shown in Supplementary Table 3. In the experiment, every 3 days, the bacteria were inoculated in nutrient agar (NA, HiMedia), MacConkey agar (MAC, HiMedia), EPM, MILi (Probac commercial kit EPM-MILi), and Simon’s citrate agar (Merck), and analyzed by Gram staining to ensure that there was no contamination.

### Statistical Method

The results were analyzed using R Statistical Software (version 3.5.1) by non-parametric tests (Wilcoxon–Mann–Whitney and Kruskal–Wallis followed by Dunn’s *post hoc* method). A *p*-value equal or less than 0.05 (*p* ≤ 0.05) was considered statistically significant.

## Results

### Characterization of the Biogenically Synthesized Silver Nanoparticles

**FIGURE 1:**
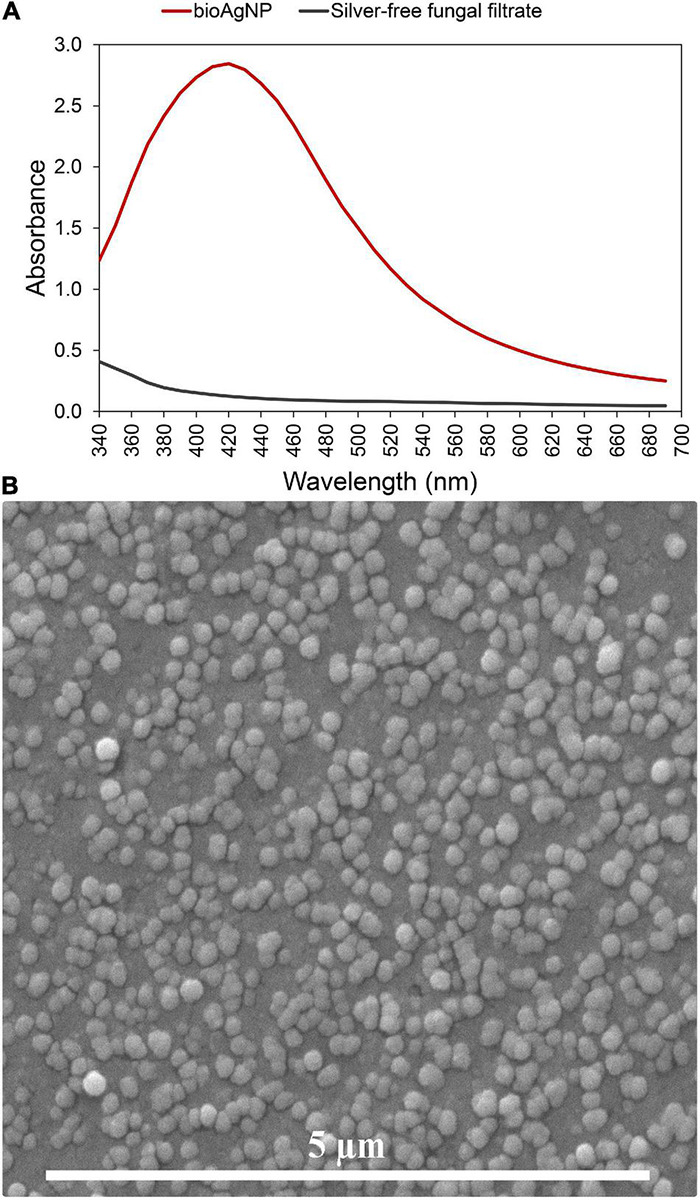
Characterization of the biogenically synthesized silver nanoparticles (bioAgNP) regarding their plasmonic band and morphology. **(A)** UV-Vis spectra of the bioAgNP and fungal-free solution. **(B)** Scanning electron microscopy (SEM) micrograph of the bioAgNP.

A fungal-free solution had a pale-yellow color before adding AgNO_3_. Immediately after adding silver salt, the color of the solution changed to brownish, and its color intensity increased after over 15 days of incubation at 28°C. The strong plasmon resonance of bioAgNP is centered at 420 nm, while the fungal-free solution (without AgNO_3_) that was used as negative control showed no absorption peak at this wavelength (Figure 1A). This change in color (from transparent to brown) and solution absorption peak centered at 420 nm (plasmonic band, showed by Figure 1) indicated that the bioAgNP were synthesized. Photon correlation spectroscopy and SEM analysis confirmed nanoparticle formation. Energy dispersive X-ray fluorescence spectrometer analysis confirmed the presence of Ag in the nanoparticle samples. Average bioAgNP diameter and zeta potential were 73.1 ± 0.5 nm and −24.2 ± 2.1 mV, respectively. Nanoparticle size (Supplementary Figure 1) and zeta potential (Supplementary Figure 2) distributions are shown in Supplementary Material. SEM analysis of the bioAgNP shows spherical nanoparticles (Figure 1B).

### Minimum Inhibitory Concentration of Oregano-Derived and Silver-Based Antibacterials Separately

All the oregano-derived compounds (OEO, Car, and Thy) and silver-based compounds (bioAgNP and AgNO_3_) inhibited the growth of all bacterial species tested in this study, including multidrug-resistant strains (Table 1). The mean MIC and MBC (inside parentheses) were 0.9 ± 0.6 mg/ml (0.9 ± 0.6 mg/ml) for OEO, 0.6 ± 0.3 mg/ml (0.7 ± 0.4 mg/ml) for Car, 0.5 ± 0.2 mg/ml (0.5 ± 0.3 mg/ml) for Thy, 15.7 ± 9.1 μg/ml (20.5 ± 7.6 μg/ml) for the bioAgNP, and 18.1 ± 10.9 μg/ml (22.3 ± 11.7 μg/ml) for AgNO_3_. For the set of bacteria tested, no statistically significant difference was found among the three oregano-based treatments (OEO, Car, and Thy) with regard to their MIC values (*p* > 0.05, Kruskal–Wallis test). There was also no statistical difference between bioAgNP and AgNO_3_ MIC mean values (*p* > 0.05, Wilcoxon test).

**TABLE 1:** Mean of minimal inhibitory and bactericidal concentrations of oregano-derived antimicrobials and silver-based compounds individually.

Bacterial strains	OEO		Car		Thy		bioAgNP		AgNO_3_	
	(mg/ml)		(mg/ml)		(mg/ml)		(μg/ml)		(μg/ml)	
	MIC	MBC	MIC	MBC	MIC	MBC	MIC	MBC	MIC	MBC
*E. coli* ATCC 25922	0.3	0.3	0.31	0.31	0.25	0.25	15.75	15.75	42.5	42.50
*E. coli* KPC 126	0.59	0.59	0.61	0.61	0.5	0.5	15.75	15.75	21.25	42.50
*K. pneumoniae* ATCC 10031	0.15	0.3	0.15	0.31	0.25	0.25	31.5	31.5	21.25	21.25
*K. pneumoniae* KPC 5795	0.59	0.59	0.61	0.61	0.5	0.5	31.5	31.5	21.25	21.25
*P. aeruginosa* ATCC 9027	0.59	0.59	0.61	0.61	0.5	0.5	7.88	15.75	21.25	21.25
*P. aeruginosa* CR 3400	1.19	1.19	0.61	0.61	0.5	0.5	7.88	15.75	5.31	10.63
*A. baumannii* ATCC 19606	0.59	0.59	0.31	0.31	0.5	0.5	7.88	15.75	5.31	10.63
*A. baumannii* CR 01	1.19	1.19	0.61	1.22	0.25	0.5	7.88	15.75	10.63	10.63
*S. aureus* ATCC 25923	2.38	2.38	1.22	1.22	0.5	1	15.75	15.75	21.25	21.25
MRSA N315	1.19	1.19	0.61	1.22	1	1	15.75	31.5	10.63	21.25

*MIC, minimal inhibitory concentration; MBC, minimal bactericidal concentration; OEO, oregano essential oil; Car, carvacrol; Thy, thymol; bioAgNP, biogenically synthesized silver nanoparticles; ATCC, American Type Culture Collection; KPC, Klebsiella pneumoniae carbapenemase; CR, carbapenem-resistant; MRSA, methicillin-resistant Staphylococcus aureus.*

*126, 5795, 3400, and 01 are strains number from the Laboratory of Basic and Applied Bacteriology, Universidade Estadual de Londrina, Brazil.*

With regard to MIC values, no statistically significant difference was observed between the two bacterial groups (reference and multidrug-resistant strains) in terms of susceptibility to OEO, Car, Thy, bioAgNP, and AgNO_3_ (*p* > 0.05, Wilcoxon test). The antimicrobials showed the following average MIC values for reference strains: 0.8 ± 0.9 (OEO), 0.5 ± 0.4 (Car), and 4 ± 0.1 mg/ml (Thy), and 15.7 ± 9.6 (bioAgNP) and 22.3 ± 13.2 μg/ml (AgNO_3_). The average MIC values for the multidrug-resistant strains are the following: 0.9 ± 0.3 (OEO), 0.6 ± 0 (Car), and 0.5 ± 0.3 mg/ml (Thy), and 15.7 ± 9.6 (bioAgNP) and 13.8 ± 7.1 μg/ml (AgNO_3_).

There was no statistically significant difference between both the Gram-negative and Gram-positive bacterial groups in terms of susceptibility to OEO, Car, Thy, bioAgNP, and AgNO_3_ with regard to their MIC values (*p* > 0.05, Wilcoxon test). The antimicrobials showed the following average MIC values for Gram-negative: 0.6 ± 0.4 (OEO), 0.5 ± 0.2 (Car), and 0.4 ± 0.1 mg/ml (Thy), and 15.7 ± 10.3 (bioAgNP) and 18.6 ± 12.1 μg/ml (AgNO_3_). The average MIC values for Gram-positive are the following: 1.8 ± 0.8 (OEO), 0.9 ± 0.4 (Car), and 0.7 ± 0.3 mg/ml (Thy), and 15.7 ± 0 (bioAgNP) and 15.9 ± 7.5 μg/ml (AgNO_3_).

### Antibacterial Interaction of Double-Combined Compounds

All the four binary-antimicrobial combinations inhibited the growth of bacterial strains tested in this study and exhibited synergistic, additive, or indifferent antibacterial effect; no antagonistic interaction was seen (Tables 2–5). The combined compounds showed statistically significant lower MIC values than the individual agents (*p* < 0.05, Wilcoxon test). In the combination containing OEO and bioAgNP (Table 2), average dose reductions were 75 ± 15% (*p* < 0.05) for OEO and 55 ± 11% (*p* < 0.05) for the bioAgNP. In the combination containing Car and bioAgNP (Table 3), average dose reductions were 67 ± 19% (*p* < 0.05) for Car and 60 ± 17% (*p* > 0.05) for the bioAgNP; even though there were no statistically significant differences between the individual and combined treatments in terms of bioAgNP MIC, the dose of Car was reduced. Thy plus bioAgNP (Table 4) resulted in average MIC reductions of 65 ± 18% (*p* < 0.05) for Thy and 61 ± 17% (*p* < 0.05) for the bioAgNP. In the combination composed of Car and Thy (Table 5), average MIC reductions were 62 ± 19% (*p* < 0.05) for Car and 76 ± 16% (*p* < 0.05) for Thy.

**TABLE 2:** Effect of oregano essential oil (OEO) combined with biological silver nanoparticles (bioAgNP) against five bacterial species, including reference and multidrug-resistant strains.

Bacterial strains	OEO		bioAgNP		FICI	Interaction
	MIC_combination_	FIC (fold decrease)	MIC_combination_	FIC (fold decrease)		
	(mg/ml)		(μg/ml)			
*E. coli* ATCC 25922	0.15	0.5 (2×)	3.94	0.25 (4×)	0.75	ADDITIVE
*E. coli* KPC 126	0.07	0.12 (8×)	7.88	0.5 (2×)	0.62	ADDITIVE
*K. pneumoniae* ATCC 10031	0.04	0.25 (4×)	15.75	0.5 (2×)	0.75	ADDITIVE
*K. pneumoniae* KPC 5795	0.15	0.25 (4×)	15.75	0.5 (2×)	0.75	ADDITIVE
*P. aeruginosa* ATCC 9027	0.59	1(ND)	7.88	1 (ND)	2	INDIFFERENT
*P. aeruginosa* CR 3400	1.19	1 (ND)	7.88	1 (ND)	2	INDIFFERENT
*A. baumannii* ATCC 19606	0.59	1 (ND)	7.88	1 (ND)	2	INDIFFERENT
*A. baumannii* CR 01	1.19	1 (ND)	7.88	1 (ND)	2	INDIFFERENT
*S. aureus* ATCC 25923	0.3	0.12 (8×)	7.88	0.5 (2×)	0.62	ADDITIVE
MRSA N315	1.19	1 (ND)	15.75	1 (NF)	2	INDIFFERENT

*Minimal inhibitory concentrations (MIC) of both OEO and bioAgNP in combination and their antibacterial interaction that is defined by fractional inhibitory concentration index (FICI) are indicated. Fold decrease describes how much the MIC of OEO and bioAgNP in combination was reduced in comparison to the MIC of the same compounds alone (absolute MIC values of compounds individually are shown in Table 1).*

*FICI, which depends on the fractional inhibitory concentration (FIC) of each compound, was interpreted below as follows:*

*≤0.5 (synergy); >0.5 to ≤1 (addition); >1 to ≤2 (indifference); >2 (antagonism). FIC value was determined using the following formula:*

*FIC = MIC_combination_/MIC_individual_.*

*FICI value was determined using the following formula: FICI = FIC_OEO_ + FIC_bioAgNP_.*

*ND, no decrease, since the MIC values of compound alone and in combination are same.*

*ATCC, American Type Culture Collection; KPC, K. pneumoniae carbapenemase; CR, carbapenem-resistant; MRSA, methicillin-resistant S. aureus.*

*126, 5795, 3400, and 01 are strain numbers from the Laboratory of Basic and Applied Bacteriology, Universidade Estadual de Londrina, Brazil.*

**TABLE 3:** Effect of carvacrol (Car) combined with biological silver nanoparticles (bioAgNP) against five bacterial species, including reference and multidrug-resistant strains.

Bacterial strains	Car		bioAgNP		FICI	Interaction
	MIC_combination_	FIC (fold decrease)	MIC_combination_	FIC (fold decrease)		
	(mg/ml)		(μg/ml)			
*E. coli* ATCC 25922	0.15	0.5 (2×)	3.94	0.25 (4×)	0.75	ADDITIVE
*E. coli* KPC 126	0.08	0.12 (8×)	7.88	0.5 (2×)	0.62	ADDITIVE
*K. pneumoniae* ATCC 10031	0.08	0.5 (2×)	15.75	0.5 (2×)	1	ADDITIVE
*K. pneumoniae* KPC 5795	0.15	0.25 (4×)	15.75	0.5 (2×)	0.75	ADDITIVE
*P. aeruginosa* ATCC 9027	0.61	1 (ND)	7.88	1 (ND)	2	INDIFFERENT
*P. aeruginosa* CR 3400	0.61	1 (ND)	7.88	1 (ND)	2	INDIFFERENT
*A. baumannii* ATCC 19606	0.31	1 (ND)	7.88	1 (ND)	2	INDIFFERENT
*A. baumannii* CR 01	0.31	0.5 (2×)	0.98	0.12 (8×)	0.62	ADDITIVE
*S. aureus* ATCC 25923	0.15	0.12 (8×)	7.88	0.5 (2×)	0.62	ADDITIVE
MRSA N315	0.61	1 (ND)	15.75	1 (ND)	2	INDIFFERENT

*Minimal inhibitory concentrations (MIC) of both Car and bioAgNP in combination and their antibacterial interaction that is defined by FICI are indicated. Fold decrease describes how much the MIC of Car and bioAgNP in combination was reduced in comparison to the MIC of same compounds alone (absolute MIC values of the compounds individually are shown in Table 1).*

*Fractional inhibitory concentration index (FICI), which depends on the fractional inhibitory concentration (FIC) of each compound, was interpreted below as follows:*

*≤0.5 (synergy); >0.5 to ≤1 (addition); >1 to ≤2 (indifference); >2 (antagonism).*

*FIC value was determined using the following formula: FIC = MIC_combination_/MIC_individual_.*

*FICI value was determined using the following formula: FICI = FIC_Car_ + FIC_bioAgNP_.*

*ND, no decrease, since the MIC values of compound alone and in combination are same.*

*ATCC, American Type Culture Collection; KPC, K. pneumoniae carbapenemase; CR, carbapenem-resistant; MRSA, methicillin-resistant S. aureus.*

*126, 5795, 3400, and 01 are strains numbers from Laboratory of Basic and Applied Bacteriology, Universidade Estadual de Londrina, Brazil.*

**TABLE 4:** Effect of thymol (Thy) combined with biological silver nanoparticles (bioAgNP) against five bacterial species, including reference and multidrug-resistant strains.

Bacterial strains	Thy		bioAgNP		FICI	Interaction
	MIC_combination_	FIC (fold decrease)	MIC_combination_	FIC (fold decrease)		
	(mg/ml)		(μg/ml)			
*E. coli* ATCC 25922	0.06	0.25 (4×)	7.88	0.5 (2×)	0.75	ADDITIVE
*E. coli* KPC 126	0.12	0.25 (4×)	7.88	0.5 (2×)	0.75	ADDITIVE
*K. pneumoniae* ATCC 10031	0.03	0.12 (8×)	15.75	0.5 (2×)	0.62	ADDITIVE
*K. pneumoniae* KPC 5795	0.06	0.12 (8×)	15.75	0.5 (2×)	0.62	ADDITIVE
*P. aeruginosa* ATCC 9027	0.25	0.5 (2×)	0.98	0.12 (8×)	0.62	ADDITIVE
*P. aeruginosa* CR 3400	0.25	0.5 (2×)	3.94	0.5 (2×)	1	ADDITIVE
*A. baumannii* ATCC 19606	0.25	0.5 (2×)	0.98	0.12 (8×)	0.62	ADDITIVE
*A. baumannii* CR 01	0.25	1 (ND)	7.88	1 (ND)	2	INDIFFERENT
*S. aureus* ATCC 25923	0.06	0.12 (8×)	7.88	0.5 (2×)	0.62	ADDITIVE
MRSA N315	0.5	0.5 (2×)	3.94	0.25 (4×)	0.62	ADDITIVE

*Minimal inhibitory concentrations (MIC) of both Thy and bioAgNP in combination and their antibacterial interaction that is defined by FICI are indicated. Fold decrease describes how much the MIC of Thy and bioAgNP in combination was reduced in comparison to the MIC of same compounds alone (absolute MIC values of the compounds individually are shown in Table 1).*

*Fractional inhibitory concentration index (FICI), which depends on the fractional inhibitory concentration (FIC) of each compound, was interpreted below as follows:*

*≤0.5 (synergy); >0.5 to ≤1 (addition); >1 to ≤2 (indifference); >2 (antagonism).*

*FIC value was determined using the following formula: FIC = MIC_combination_/MIC_individual_.*

*FICI value was determined using the following formula: FICI = FIC_Thy_ + FIC_bioAgNP_.*

*ND, no decrease, since the MIC values of compound alone and in combination are same.*

*ATCC, American Type Culture Collection; KPC, K. pneumoniae carbapenemase; CR, carbapenem-resistant; MRSA, methicillin-resistant S. aureus.*

*126, 5795, 3400, and 01 are strain numbers from the Laboratory of Basic and Applied Bacteriology, Universidade Estadual de Londrina, Brazil.*

**TABLE 5:** Effect of carvacrol (Car) combined with thymol (Thy) against five bacterial species, including reference and multidrug-resistant strains.

Bacterial strains	Car		Thy		FICI	Interaction
	MIC_combination_	FIC (fold decrease)	MIC_combination_	FIC (fold decrease)		
	(mg/ml)		(mg/ml)			
*E. coli* ATCC 25922	0.15	0.5 (2×)	0.03	0.12 (8×)	0.62	ADDITIVE
*E. coli* KPC 126	0.31	0.5 (2×)	0.12	0.25 (4×)	1	ADDITIVE
*K. pneumoniae* ATCC 10031	0.02	0.12 (8×)	0.12	0.5 (2×)	0.62	ADDITIVE
*K. pneumoniae* KPC 5795	0.31	0.5 (2×)	0.06	0.12 (8×)	0.62	ADDITIVE
*P. aeruginosa* ATCC 9027	0.08	0.12 (8×)	0.25	0.5 (2×)	0.62	ADDITIVE
*P. aeruginosa* CR 3400	0.61	1 (ND)	0.5	1 (ND)	2	INDIFFERENT
*A. baumannii* ATCC 19606	0.15	0.5 (2×)	0.12	0.25 (4×)	0.75	ADDITIVE
*A. baumannii* CR 01	0.31	0.5 (2×)	0.03	0.12 (8×)	0.75	ADDITIVE
*S. aureus* ATCC 25923	0.61	0.5 (2×)	0.06	0.12 (8×)	0.75	ADDITIVE
MRSA N315	0.08	0.12 (8×)	0.12	0.12 (8×)	0.25	SYNERGISM

*Minimal inhibitory concentrations (MIC) of both Car and Thy in combination and their antibacterial interaction that is defined by FICI are indicated. Fold decrease describes how much the MIC of Car and Thy in combination was reduced in comparison to the MIC of same compounds alone (absolute MIC values of the compounds individually are shown in Table 1).*

*Fractional inhibitory concentration index (FICI), which depends on the fractional inhibitory concentration (FIC) of each compound, was interpreted below as follows:*

*≤0.5 (synergy); >0.5 to ≤1 (addition); >1 to ≤2 (indifference); >2 (antagonism).*

*FIC value was determined using the following formula: FIC = MIC_combination_/MIC_individual_.*

*FICI value was determined using the following formula: FICI = FIC_Car_ + FIC_Thy_.*

*ND, no decrease, since the MIC values of compound alone and in combination are same.*

*ATCC, American Type Culture Collection; KPC, K. pneumoniae carbapenemase; CR, carbapenem-resistant; MRSA, methicillin-resistant S. aureus.*

*126, 5795, 3400, and 01 are strain numbers from the Laboratory of Basic and Applied Bacteriology, Universidade Estadual de Londrina, Brazil.*

### Time-Kill Curve

**FIGURE 2:**
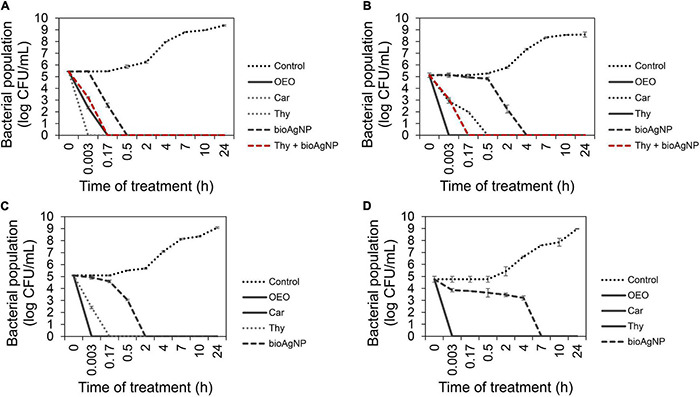
Time-kill curves of Gram-negative and Gram-positive bacterial strains exposed to oregano essential oil (OEO), carvacrol (Car), thymol (Thy), and the bioAgNP individually at minimal bactericidal concentration (MBC), and in the combination containing Thy (0.5× MIC) and bioAgNP (0.5× minimal inhibitory concentration, MIC). Control indicates bacterial growth with no antimicrobial. **(A)** *Escherichia coli* ATCC 25922. **(B)** KPC-producing *Klebsiella pneumoniae*. **(C)** Carbapenem-resistant *Acinetobacter baumannii*. **(D)** Methicillin-resistant *Staphylococcus aureus* (MRSA) N315. Values of log10 colony-forming units (CFU)/ml are the mean ± standard deviation.

For each oregano-derived antimicrobial, comparative analysis among nine treatment times showed statistically significant difference in terms of CFU/ml (*p* < 0.05, Kruskal–Wallis test); OEO, Car, and Thy caused a very fast reduction in the number of CFU/ml of all the tested four strains: *E. coli* ATCC 25922 (Figure 2A), KPC-producing *K. pneumoniae* (Figure 2B), carbapenem-resistant *A. baumanni*i (Figure 2C), and MRSA N315 (Figure 2D).

After immediate treatment (10 s), OEO reduced bacterial population by approximately 5.1 ± 0.2 log (*p* < 0.05) for KPC-producing *K. pneumoniae* (Figure 2B), 5.1 ± 0.04 log (*p* < 0.05) for carbapenem-resistant *A. baumannii* (Figure 2C), and 4.8 ± 0.2 log (*p* < 0.05) for MRSA N315 (Figure 2D). OEO decreased *E. coli* ATCC 25922 population (Figure 2A) by 3.1 ± 0.1 log (*p* < 0.05) and 5.4 ± 0.02 log (*p* < 0.05) after 10-s and 10-min treatments, respectively.

After 10 s of Car treatment, bacterial populations reduced approximately by 2.2 ± 0.05 log (*p* < 0.05) for KPC-producing *K. pneumoniae*, 5.4 ± 0.02 log (*p* < 0.05) for standard *E. coli* (Figure 2A), 5.1 ± 0.04 log (*p* < 0.05) for carbapenem-resistant *A. baumannii* (Figure 2C), and 4.8 ± 0.2 log (*p* < 0.05) for MRSA N315 (Figure 2D). Car decreased carbapenem-resistant *K. pneumoniae* (Figure 2B) approximately by 3.1 ± 0.23 log (*p* < 0.05) and 5.1 ± 0.2 log (*p* < 0.05) after 10- and 30-min treatments, respectively.

After 10 s, Thy reduced bacterial population by approximately 5.4 ± 0.02 log (*p* < 0.05) for *E. coli* (Figure 2A), 5.1 ± 0.2 log (*p* < 0.05) for carbapenem-resistant *K. pneumoniae* (Figure 2B), and 4.8 ± 0.2 log (*p* < 0.05) for MRSA N315 (Figure 2D). The population of carbapenem-resistant *A. baumannii* (Figure 2C) was reduced by 2.7 ± 0.1 log (*p* < 0.05) and 5.1 ± 0.04 log (*p* < 0.05) after 10 s and 10 min of exposition to Thy, respectively.

For bioAgNP-treatment, a comparative analysis among nine treatment times showed statistically significant difference in terms of CFU/ml (*p* < 0.05, Kruskal–Wallis test). *E. coli* ATCC 25922 (Figure 2A) was reduced by the bioAgNP by approximately 2.9 ± 0.2 log (*p* < 0.05) and 5.4 ± 0.02 log (*p* < 0.05) after 10 and 30 min of treatment, respectively. KPC-producer *K. pneumoniae* (Figure 2B) was reduced by the bioAgNP by about 2.8 ± 0.1 log (*p* < 0.05) and 5.1 ± 0.2 log (*p* < 0.05) after 2 and 4 h, respectively. Carbapenem-resistant *A. baumannii* (Figure 2C) was reduced by the bioAgNP by about 2 ± 0.04 log (*p* < 0.05) and 5.1 ± 0.04 log (*p* < 0.05) after 30 min and 2 h, respectively. MRSA N315 (Figure 2D) was reduced by the bioAgNP by around 4.8 ± 0.2 log (*p* < 0.05) after 7 h of treatment.

For the combination treatment composed of Thy and bioAgNP, comparative analysis among nine treatment times showed statistically significant difference in terms of CFU/ml (*p* < 0.05, Kruskal–Wallis test). Both combined compounds caused a faster reduction of Enterobacteriaceae strains in CFU/ml than the individual treatment with bioAgNP. After 10 s of treatment, Thy plus bioAgNP (both at 0.5× individual MIC) decreased *E. coli* ATCC 25922 (Figure 2A) by 2.2 ± 0.1 log (*p* < 0.05) and KPC-producing *K. pneumoniae* (Figure 2B) by 2 ± 0.1 log (*p* < 0.05); both bacterial populations resulted in no detected viable cells (approximately 5.1 ± 0.2 log reduction, *p* < 0.05) after 10 min of exposition to the binary antimicrobial combination.

### Effect of the Antimicrobials on Bacterial Intracellular Reactive Oxygen Species

**FIGURE 3:**
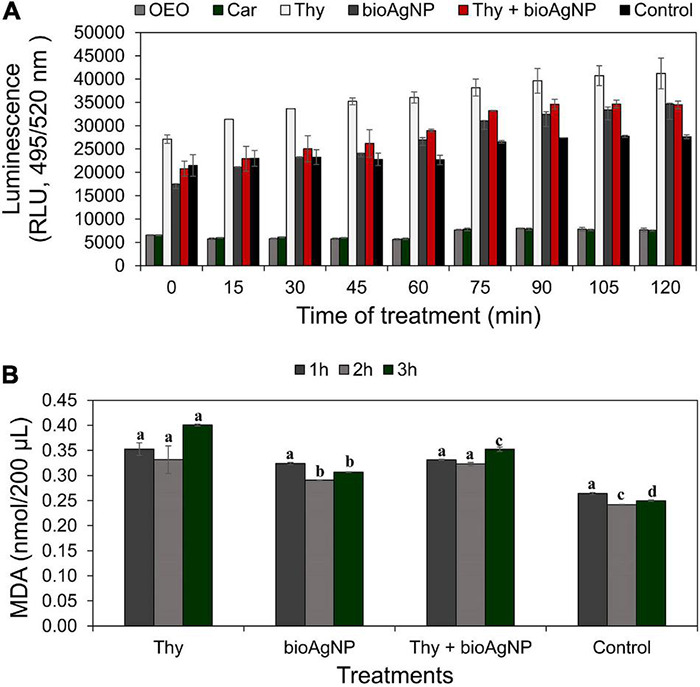
Measurement of oxidative stress over time in *E. coli* ATCC 25922 exposed to antimicrobials at subinhibitory concentrations (oregano-derived antimicrobials and the bioAgNP individually and in combination) by quantification of intracellular reactive oxygen species (ROS) generation and lipid peroxidation levels. Controls indicate bacterial ROS and malondialdehyde (MDA) generation by cells without the antimicrobials. **(A)** ROS production levels expressed as relative light units (RLU) whose values are the mean ± standard deviation. **(B)** Lipid peroxidation levels measured by malondialdehyde (MDA) production after 1, 2, and 3 h of treatment. Values of MDA are the mean ± standard deviation. ^a–d^ Indicate statistically significant difference (*p* < 0.05, Kruskal–Wallis test) among treatments and control at the same time in term of oxidative stress; different letters indicate difference, and same letters indicate absence of difference.

Figure 3A shows ROS production levels by treated and non-treated *E. coli* ATCC 25922, expressed as luminescent values (RLU, relative light units, using excitation and emission wavelengths of 495 nm and 520 nm, respectively, based on untreated control which showed natural production of ROS). For all the treatment time points (0–2 h), even though there were no statistically significant differences among OEO, Car, Thy, bioAgNP, combination of Thy plus bioAgNP, and control in terms ROS production (*p* > 0.05, Kruskal–Wallis test), three treated bacterial samples (Thy, bioAgNP, and Thy plus bioAgNP) presented higher levels of ROS than the untreated control in absolute values. In this study, the small size of the samples is a limiting factor for statistical statement; however, the difference in absolute numbers may be biologically relevant. Thy, bioAgNP, and their combination increased ROS levels (in absolute values) compared to the untreated control as follows: increase of 45% for the Thy-treated sample during 2 h of exposition to the antimicrobial (0–2 h); 20% for the bioAgNP (1–2 h), and 26% for the combination containing Thy and bioAgNP (1–2 h). The OEO and Car-treated bacterial samples showed lower levels of ROS (in absolute values) than the untreated control. Individually, both OEO and Car reduced ROS levels by 73% compared to the control (during 2 h of treatment).

### Effect of the Antimicrobials on Membrane Lipid Peroxidation of Bacteria

Malondialdehyde production of treated and untreated *E. coli* ATCC 25922 is expressed in nmol of MDA per 200 μl (Figure 3B). Non-treated *E. coli* showed natural production of MDA. After both 2 and 3 h of treatment, comparative analysis among the four treatments (Thy, bioAgNP, combination of both, and untreated control) showed a statistically significant difference in terms of MDA production (*p* < 0.05, Kruskal–Wallis test). After 2 h, the amount of MDA increased by 37 (Thy), 20 (bioAgNP), and 34% (combination of Thy and bioAgNP). After 3-h treatment, percentage increases were 60, 23, and 41%, respectively. Thy treatment caused significantly (*p* < 0.05) more lipid peroxidation than the other two treatments. MDA production arrangement from highest to lowest is as follows: Thy, combination, and bioAgNP (data related to 3 h of treatment).

### Bacterial Extracellular ATP Levels

**FIGURE 4:**
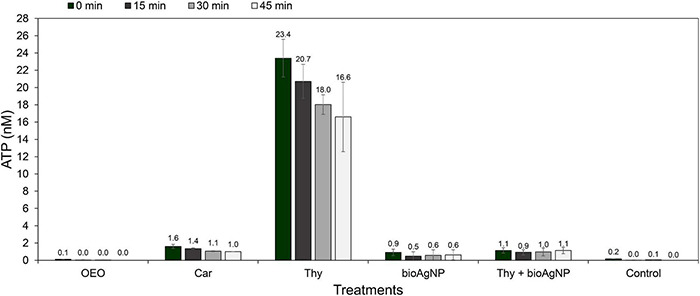
Extracellular ATP concentrations over the time of*E. coli* ATCC 25922 exposed to the oregano-derived antimicrobials and bioAgNP individually and in combination at subinhibitory doses. Control indicates ATP leakage from untreated bacterial sample. ATP levels were measured at four time points of treatment: 0-h time point, after 15 min of treatment, after 30 min of treatment, and after 45 min of treatment. Values of ATP (nM) are the mean ± standard deviation.

For the four tested time points (0–45 min), even though there were no statistically significant differences among OEO, Car, Thy, bioAgNP, Thy plus bioAgNP, and untreated control in terms of extracellular ATP amount (*p* > 0.05, Kruskal–Wallis test), all the antimicrobials increased *E. coli* ATCC 25922 ATP leakage (in absolute values, which are biologically relevant) compared to the bacterial control (Figure 4). At the 0-h time point, the amounts of extracellular ATP were 0.7-fold (OEO), 9-fold (Car), 135-fold (Thy), 5-fold (bioAgNP), and 6-fold (Thy plus AgNPs) greater than the untreated control. At 15|30|45 min of treatment, the amounts of extracellular ATP were, respectively, 2|0.4|0-fold (OEO), 107|19|1-fold (Car), 1,633|330|17-fold (Thy), 39|10|0.6 (bioAgNP), and 73|18|1-fold (Thy plus bioAgNP) greater than the untreated control.

### Effect of the Antimicrobials on Membrane Leakage of Biomolecules

**FIGURE 5:**
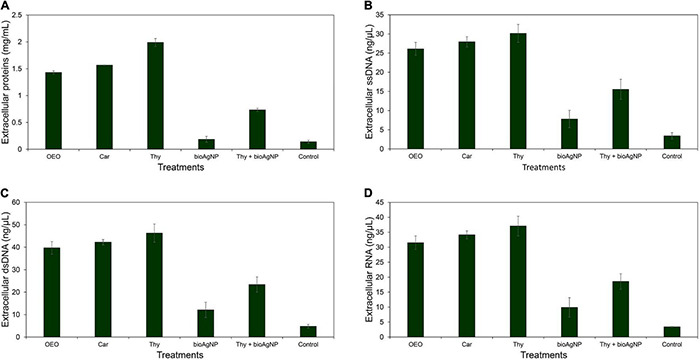
Extracellular protein, DNA and RNA concentrations of *E. coli* ATCC 25922 exposed for 1 h to the oregano-derived antimicrobials and bioAgNP individually and in combination at subinhibitory concentrations. Control indicates extracellular biomolecule concentration of bacterial cells without the antimicrobials. **(A)** Extracellular total proteins (mg/ml). **(B)** Extracellular single-stranded DNA (ssDNA; ng/μl). **(C)** Extracellular double-stranded DNA (dsDNA; ng/μl). **(D)** Extracellular RNA (ng/μl).

Even though there were no statistically significant differences among OEO, Car, Thy, bioAgNP, Thy plus bioAgNP, and untreated control in terms of cellular biomolecular leakage (total proteins, ssDNA, dsDNA, and RNA) (*p* > 0.05, Kruskal–Wallis test), all the treatments, mainly the oregano-derived compounds and the combination of Thy and bioAgNP, caused greater leakage amount of biomolecules than the untreated control *E. coli* ATCC 25922 (Figures 5A–D). Total protein leakages were 10-fold (OEO), 11-fold (Car), 14-fold (Thy), 1-fold (bioAgNP), and 5-fold (Thy plus AgNP) greater than the untreated control. For nucleic acids, ssDNA|dsDNA|RNA, the amounts of leakage were, respectively, 8|8|9-fold (OEO), 8|9|10-fold (Car), 9|10|11-fold (Thy), 2|2|3-fold (bioAgNP), and 4|5|5-fold (Thy plus bioAgNP) greater than the untreated control.

### Electron Microscopy Study of Bacteria

**FIGURE 6:**
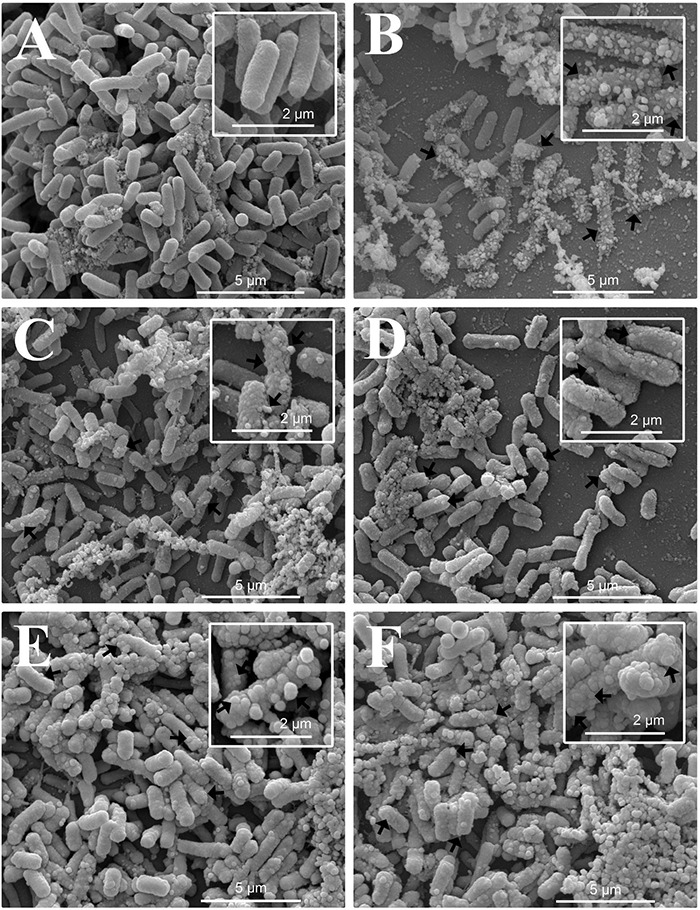
Scanning electron micrographs of the antibacterial effect of the oregano-derived antimicrobials and bioAgNP individually and in combination against *E. coli* ATCC 25922. Bacteria were exposed for 30 min to subinhibitory concentrations of the antimicrobials. **(A)** Untreated control. **(B)** OEO-treated cells. **(C)** Car-treated cells. **(D)** Thy-treated cells. **(E)** BioAgNP-treated cells. **(F)** Bacterial cells treated with the combination of Thy plus bioAgNP. Micrographs **(A–F)** show bacterial cell density, size, shape, and surface morphological changes (15,000×). Inset images show in detail the morphological alterations of treated cells and typical cells of the untreated control (30,000×). Arrows: morphological changes (surface protrusions), cellular debris and size-changed cells.

**FIGURE 7:**
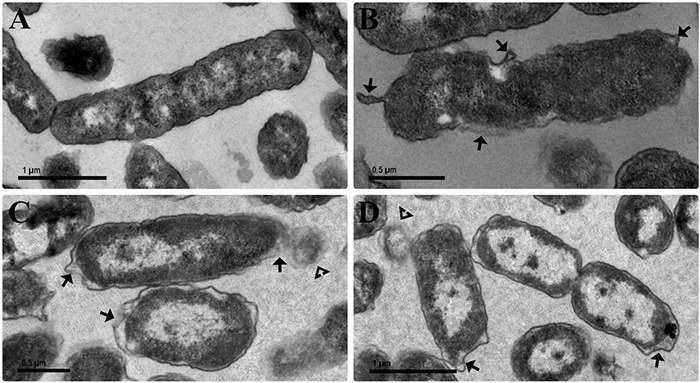
Transmission electron micrographs of the antibacterial effect of the bioAgNP individually and in combination against *E. coli* ATCC 25922. Bacteria were exposed for 1 h to the bioAgNP at subinhibitory concentration. **(A)** Untreated control showing no changes in cell morphology and regular electron density. **(B)** BioAgNP-treated cells with morphological changes. **(C,D)** BioAgNP-treated cells with disruption of external ultrastructures and reduced electron density. Arrow: damaged cellular wall and cytoplasmic membrane. Arrowheads: leakage of cytoplasmic contents.

Figures 6A–F show scanning electron micrographs of the effect of OEO, Car, Thy, bioAgNP, and the combination of Thy and bioAgNP against *E. coli* ATCC 25922. The untreated bacterial sample showed typical size and rod-shaped cells after 30 min of incubation (Figure 6A). Inset images (higher magnification) show intact surface of standard *E. coli* (Figure 6A, inset). OEO-treated cells were damaged (surface protrusions), with no typical size of bacterial species, and their population showed a slightly less amount of cells compared to the untreated control (Figure 6B). The inset image shows details, in higher magnification, of morphological changes on cell surface (Figure 6B, inset). Car-treated cells were also damaged (surface protrusions), and their population presented a slightly lower density compared to the untreated control (Figure 6C). The inset image shows details (in high magnification) of cell surface damage (Figure 6C, inset). Treatment with Thy showed cell density decrease and caused similar surface protrusions (Figure 6D). The inset shows, in higher magnification, morphological surface changes (Figure 6D, inset). The bioAgNP-treated sample showed very deformed cells, with no typical size and shape of *E. coli*, and damages appeared as cell surface blebbing (Figure 6E). The inset image shows details, in higher magnification, of cell injuries (Figure 6E, inset). MET study (Figure 7) showed that bioAgNP alone damaged cellular wall and cytoplasmic membrane. Cells treated with the combination of Thy and bioAgNP (Figure 6F) were quite deformed (surface protrusions) and had no *E. coli* common size; such changes are shown in detail in the inset image. Slight cell density decrease was observed in the OEO-, Car-, and Thy-treated samples compared to the untreated control. This reduction does not represent decrease in viable cells amount, since concentrations of the antimicrobials were subinhibitory; possibly it is the result of cell loss during the preparation of samples for microscopy analysis, such as the multiple washing steps.

### Cytotoxicity in Human Erythrocytes

**FIGURE 8:**
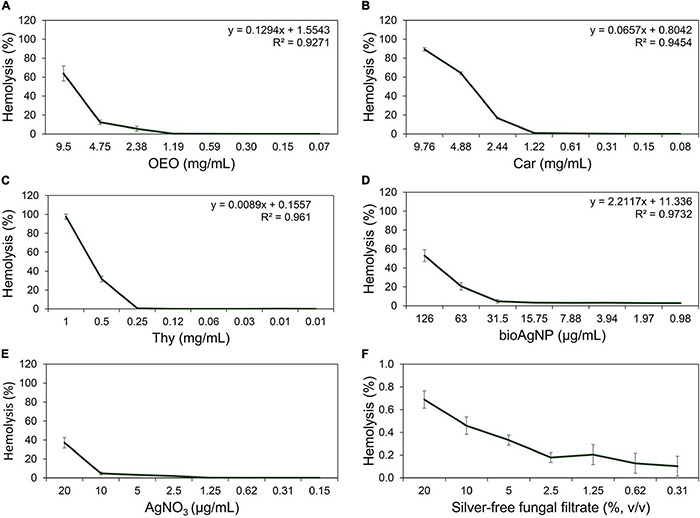
Hemolytic activity at different concentrations of the oregano-derived antimicrobials and silver-based compounds individually. **(A)** OEO. **(B)** Car. **(C)** Thy. **(D)** BioAgNP. **(E)** AgNO_3_. **(F)** *F. oxysporum*-free solution used in bioAgNP synthesis. Maximum tested concentrations of the fungal-free solution and bioAgNP have equivalent volumes. Values of hemolysis percentage are the mean ± standard deviation. The linear model equation used to predict the CC_50_ of each antimicrobial and its R-squared (*R*^2^) are shown.

Antimicrobial CC_50_ in red blood cells (RBC) were 8 (OEO, Figure 8A), 4 (Car, Figure 8B), and 0.6 mg/ml (Thy, Figure 8C), and 121.9 (bioAgNP, Figure 8D) and >170 μg/ml (AgNO_3_; Figure 8E). The *F. oxysporum*-free solution showed extremely low hemolytic activity even at highest tested concentration (Figure 8F).

All the four double antibacterial combinations (OEO plus bioAgNP, Car plus bioAgNP, Thy plus bioAgNP, and Car plus Thy) showed a slightly higher activity to erythrocytes compared to individual compounds (Table 6). All the combined treatments showed no CC_50_ in RBC at tested concentrations, especially at MIC.

**TABLE 6:** Hemolytic activity of the four binary antibacterial combinations (OEO plus bioAgNP, Car plus bioAgNP, Thy plus bioAgNP, and Car plus Thy).

Binary-antibacterial combinations			
OEO (mg/ml)	Plus	bioAgNP (μg/ml)	Hemolysis (%)
1.19	+	15.75	12.5 ± 3.2 ^NC^
0.59	+	7.88	11.4 ± 4.2 ^NC^
0.30	+	3.94	0 ± 0 ^NC^
0.15	+	1.97	0 ± 0 ^NC^
**Car (mg/ml)**	**Plus**	**bioAgNP (μg/ml)**	**Hemolysis (%)**
0.61	+	7.88	23.4 ± 2.7 ^NC^
0.31	+	3.94	5.1 ± 2.3 ^NC^
0.15	+	1.97	0 ± 0 ^NC^
0.08	+	0.98	0 ± 0 ^NC^
**Thy (mg/ml)**	**Plus**	**bioAgNP (μg/ml)**	**Hemolysis (%)**
0.5	+	15.75	39.9 ± 4.6 ^NC^
0.25	+	7.88	16.5 ± 4.9 ^NC^
0.12	+	3.94	0 ± 0 ^NC^
0.06	+	1.97	0 ± 0 ^NC^
**Car (mg/ml)**	**Plus**	**Thy (mg/ml)**	**Hemolysis (%)**
0.61	+	0.25	31.2 ± 6.2 ^NC^
0.31	+	0.12	1.9 ± 0.5 ^NC^
0.15	+	0.06	0 ± 0 ^NC^
0.08	+	0.03	0 ± 0 ^NC^

*The hemolysis percentage of each binary compound treatment in four different concentrations is indicated.*

*NC, non-cytotoxic; ±, standard deviation; OEO, oregano essential oil; Car, carvacrol; Thy, thymol; bioAgNP, biogenically synthesized silver nanoparticles.*

### Prolonged Exposure of *Escherichia coli* to the Antimicrobials

Results of initial studies on prolonged exposure to antimicrobials are shown in Supplementary Table 3. *E. coli* grew in the presence of bioAgNP MIC after 12 days of prolonged treatment; since then, this microorganism was no longer susceptible to bioAgNP concentrations that were gradually increased. After 25 days of bioAgNP treatment, mean MIC value was >16-fold greater than before starting the experiment, changing from 15.7 to >252 μg/ml. After prolonged treatment with Thy individually or combined to bioAgNP, *E. coli* did not change its sensitivity, since the mean MIC values were same before and after the experiment; 0.25 mg/ml for Thy, and 0.12 mg/ml and 7.9 μg/ml for Thy and bioAgNP in combination, respectively. The sample that received combination-prolonged treatment also did not develop tolerance to bioAgNP. The bacterial samples did not show changes (after prolonged exposure to all the antimicrobials) with regard to biochemical characteristics, colony morphology, and response to Gram-staining according the following test results: positive for glucose fermentation, gas production, bacterial movement, production of lysine decarboxylase, indole production, lactose fermentation; negative for hydrogen sulfide production, presence of urease and L-tryptophan deaminase, citrate utilization, and negative Gram-staining.

## Discussion

The results of this study highlight the powerful action of *F. oxysporum*-bioAgNP combined to Car or Thy against Gram-negative bacteria, including carbapenem-resistant strains such as *E. coli*, *K. pneumoniae*, *A. baumannii*, and *P. aeruginosa* that have become a major concern in hospitals worldwide. These bioAgNP, combined with the oregano derivatives, may prevent the emergence of bacterial resistance besides expanding the application of these terpenoids, since additive interaction reduces their strong organoleptic effects.

This research showed that the oregano-derived antimicrobials have potent bactericidal activity at low doses, in agreement with previous studies, but with slight variations in MIC and time of action due to differences in OEO composition and purification processes of Car and Thy (de Barros et al., 2009; Magi et al., 2015; Scandorieiro et al., 2016; Wijesundara and Rupasinghe, 2018; Sim et al., 2019; Ebani et al., 2020). As can be seen in Table 1, MIC values range from 0.15 to 2.38 mg/ml for OEO, 0.15 to 1.22 mg/ml for Car, and 0.25 to 1 mg/ml for Thy. Furthermore, the oregano-derived compounds were bactericidal against all the tested multidrug-resistant strains. OEO MBC values were 0.59 mg/ml against KPC-producing Enterobacteriaceae (*E. coli* and *K. pneumoniae*), and 1.19 mg/ml against the carbapenem-resistant non-Enterobacteriaceae strains (*A. baumannii* and *P. aeruginosa*) and MRSA. Car MBC values were 0.61 mg/ml against carbapenem-resistant strains such as Enterobacteriaceae and *P. aeruginosa*. Thy MBC values were 0.5 mg/ml against the carbapenem-resistant Gram-negative strains (*E. coli*, *K. pneumonia*, *P. aeruginosa*, and *A. baumannii*) and 1 mg/ml for MRSA. Similar results were found by other researchers. Kozics et al. (2019) reported that OEO MIC values were 0.5 mg/ml against *K. pneumoniae* and 1.25 mg/ml for *P. aeruginosa*, which are both multidrug-resistant strains. Sim et al. (2019) reported that the MBC of oregano compounds against *P. aeruginosa* strains ranged from 0.563 to 1.173 (OEO), 0.585 to 1.12 (Car), and 0.4 to 0.8 mg/ml (Thy).

The time-kill assays confirmed that the three oregano compounds are bactericidal and have an extremely fast action (within a few seconds), corroborating other studies (de Barros et al., 2009; Scandorieiro et al., 2016; Wijesundara and Rupasinghe, 2018; Sim et al., 2019). Figure 2 shows that OEO, Car, and Thy reduced bacterial population by approximately 5 log after at least 10 s of treatment. There were no detected viable cells after 10 s of exposition to OEO (carbapenem-resistant strains such as *K. pneumoniae* and *A. baumannii*, and MRSA), Car (reference *E. coli*, carbapenem-resistant *A. baumannii*, and MRSA), and Thy (reference *E. coli*, KPC-producing *K. pneumoniae*, and MRSA). Sim et al. (2019) showed that OEO, Car, and Thy reduced approximately 6.5 log of *P. aeruginosa* cells after 1 h of treatment. The Wijesundara and Rupasinghe (2018) research showed that OEO caused 99.9% elimination of an initial *Streptococcus pyogenes* inoculum after 5 min of exposure.

The antimicrobial activity of essential oils may not rely exclusively on their main constituents, but it results from interactions among main compounds and minor components such as phenols, aldehydes, ketones, alcohols, esters, ethers, hydrocarbons, and others (Bassolé and Juliani, 2012; Hyldgaard et al., 2012). This study shows that the combination containing pure Car and Thy presented synergistic and additive antibacterial interactions (Table 5; Car plus Thy showed higher antibacterial activity than both compounds individually, since their combination reduced significantly the MIC values by 62% for Car and 76% for Thy, and such results are in agreement with previous studies) (Zhou et al., 2007; Pei et al., 2009; Rivas et al., 2010). However, the antibacterial activity of crude OEO alone is similar to that of pure Car or pure Thy individually (Table 1), since their average MIC values were statistically similar (*p* > 0.05). In our result, even though OEO has Car and Thy in its composition, the synergistic interaction between main components is not evident in oil activity; small amount of Thy (2%) or crude OEO trace components may interfere with oil activity and may mask the synergism between Car and Thy. Unlike what we observed in our results, some studies reported that trace components in crude oil may help in its activity, showing that the oil presents higher antimicrobial activity than the main components (individually or in combination) or synergistic interaction between p-cymene and Car (Lambert et al., 2001; Burt, 2004; Rattanachaikunsopon and Phumkhachorn, 2010; Hyldgaard et al., 2012). Some studies suggest that minor components may cause antagonistic interactions, since main components alone are more effective than crude oil (Rao et al., 2010; Hyldgaard et al., 2012; Magi et al., 2015). Regarding our data, it is important to highlight that such oil trace elements were not antagonistic, since OEO did not show worse antibacterial effect than Car and Thy alone.

Some studies have found that Gram-positive strains are more sensitive to essential oil, including oregano derivatives. It may happen because such oils directly impair the cytoplasmic membrane, and the cell wall of bacteria lacks an outer membrane, which is a barrier that prevents easy access of hydrophobic molecules (Stojković et al., 2013; Sakkas and Papadopoulou, 2017; Khan et al., 2019; Sim et al., 2019). This study indicates that OEO, Car, and Thy have a broad-spectrum action in agreement with results reported by other researchers (Rosato et al., 2010; Alvarez et al., 2014; Scandorieiro et al., 2016) showing all the three compounds to have a similar activity with regard to time of action (Figure 2) and MIC (Table 1) against Gram-positive and Gram-negative, including *P. aeruginosa* whose cell wall is rich in porins. Some slight variations in antimicrobial data from different studies (with oregano compounds) may occur, because such antimicrobials are derived from plants and have variations in their chemical composition, which depend on climatic, geographical, and extraction methods, among others (Leyva-López et al., 2017; Gavahian et al., 2018). In addition, different bacterial strains used in several studies may have structural and metabolic variations; for example, some strains have more porins than others, and such differences may make them more or less sensitive to such compounds.

Despite their strong antibacterial activity, remarkable organoleptic features of oregano-derived compounds may limit their use (Alvarez et al., 2014; Kotronia et al., 2017). In order to overcome this problem, our research group proposes the association of these oregano-derived compounds with bioAgNP; undesired organoleptic effects of oregano derivatives might be reduced, since synergistic or additive combinations decrease the necessary concentration of each antimicrobial.

In this study, the bioAgNP exhibited a broad-spectrum antibacterial action (Table 1), inhibiting growth of both Gram-positive and Gram-negative bacteria, in agreement with the literature (de Lima et al., 2012; Durán et al., 2016b; Scandorieiro et al., 2016; Singh et al., 2016; Dalir et al., 2020; Sathiyaseelan et al., 2020; Urzedo et al., 2020). Our results showed that mean bioAgNP MIC (15.7 ± 9.1 μg/ml) is similar to values reported by previous studies on same nanoparticles, which were produced with *F. oxysporum* components, showing spherical shape, similar size, zeta potential, and capping agents (Cardozo et al., 2013; Marcato et al., 2013; Biasi-Garbin et al., 2015; Scandorieiro et al., 2016). With regard to MIC, the bioAgNP were equally effective against Gram-positive and negative strains; these results are in line with other studies (Ghosh et al., 2013; Cavassin et al., 2015; Singh et al., 2016). Some researchers have found that Gram-positive strains are more tolerant to these nanoparticles, since their MIC values are higher than their values against Gram-negative strains (Kim et al., 2011; Dalir et al., 2020; Sathiyaseelan et al., 2020). However, our data showed that bioAgNP time of action (Figure 2) was faster against Gram-negative, in agreement with some studies (Jain et al., 2009; Agnihotri et al., 2014; Scandorieiro et al., 2016). Our data of SEM micrographs also show that the bioAgNP cause greater surface alterations in *E. coli* (Figure 6) than in *S. aureus* (Scandorieiro et al., 2016). It was difficult to compare our results with the literature, since silver nanoparticles may vary according to many aspects that interfere in their antimicrobial activity such as size, morphology, type and presence of stabilizing agents, and surface charge (Ayala-Núñez et al., 2009; Agnihotri et al., 2014; Durán et al., 2016a; Nisar et al., 2019; Sánchez-López et al., 2020). Besides that, different studies employ distinct techniques for nanoparticle characterization and microbiological analysis, which affect the conclusion with regard to their antimicrobial activity. We highlight the importance of standardization of bioAgNP characterization and their microbiological assays (Durán et al., 2016b).

The bioAgNP and AgNO_3_ showed similar antibacterial efficiency with regard to their MIC values (Table 1). Some studies suggest that silver nanoparticles have mechanisms of action different from those of salt-derived silver ion (Despax et al., 2011; Xiu et al., 2012; Yan et al., 2018). In this study, the bioAgNP had 73 nm, on average; probably the relatively large size did not interfere with their antibacterial activity, and it may result in no toxicity (data shown in hemolytic assay). However, it is necessary to investigate how coat proteins of these bioAgNP (Durán et al., 2005) influence their antibacterial activity to know in details the mechanism of action as antibacterial.

Although the bioAgNP show low toxicity and broad antibacterial action, bacteria can easily develop resistance to these nanoparticles by simple and fast genetic changes (Losasso et al., 2014; Graves et al., 2015; Panáček et al., 2017; Muller, 2018). In this study, the data show that *E. coli* ATCC 25922 became tolerant to the bioAgNP after only 12 days of daily treatment with this nanometal; MIC value increased from 15.7 to >252 μg/ml after 25 days of daily treatment (data are shown in Supplementary Table 3). Thus, alternative studies are needed to work around bioAgNP emergence of resistance. However, *E. coli* ATCC 25922 did not develop resistance to Thy plus bioAgNP, or to the bioAgNP, after daily exposition to the combination composed of Thy and bioAgNP.

Combinatory antimicrobial therapy is recommended as a strategy to control antimicrobial resistance and extend the life of antimicrobial agents, since multiple drug treatments may disrupt many bacterial functions and reduce the selection of resistant strains (Yap et al., 2014; Suzuki et al., 2017; Tyers and Wright, 2019). Combinations containing conventional antibacterials are already practiced in clinical settings to combat resistant Gram-positive and Gram-negative strains (Eades et al., 2017; Jacobs et al., 2017; Doi, 2019). BioAgNP showed antimicrobial synergistic or additive effect when combined with several essential oils, their main constituents, and other natural compounds (Cardozo et al., 2013; Biasi-Garbin et al., 2015; Otaguiri et al., 2016; Scandorieiro et al., 2016; Dehkordi et al., 2019), and some conventional antimicrobials and other materials or drugs (Kora and Rastogi, 2013; Longhi et al., 2016; Andrade et al., 2017; Bankier et al., 2019; Bocate et al., 2019; Figueiredo et al., 2019; Vazquez-Muñoz et al., 2019; Meroni et al., 2020) such as phenazine-1-carboxamide, eugenol, oregano oil, copaiba oil, carbon dots, tungsten carbide nanoparticles, cupper nanoparticles, simvastatin, fluconazole, carbenicillin, streptomycin, ampicillin, tetracycline, kanamycin, and chloramphenicol. Other studies have also reported an antimicrobial synergistic or additive effect of oregano derivatives in combination with others natural compounds (Ayari et al., 2020; Cho et al., 2020) and several conventional antimicrobials (Hamoud et al., 2014; Langeveld et al., 2014; Yap et al., 2014; Magi et al., 2015; Xiao et al., 2020) such as cinnamon essential oil, thyme-derived Thy, cinnamon bark essential oil, erythromycin, fluoroquinolones, doxycycline, lincomycin, vancomycin, amoxicillin, gentamicin, levofloxacin, ciprofloxacin, rifampin, and polymyxin.

Our double combined compound assays showed that the four combinations (OEO plus bioAgNP, Car plus bioAgNP, Thy plus bioAgNP, and Car plus Thy) inhibited the growth of all the tested bacteria, including the multidrug-resistant strains (Tables 2–5). The combinations reduced the MIC values when compared to individual treatments, in agreement with other studies involving essential oils and/or bioAgNP (Pei et al., 2009; Rivas et al., 2010; Cardozo et al., 2013; Biasi-Garbin et al., 2015; Longhi et al., 2016; Otaguiri et al., 2016; Scandorieiro et al., 2016; Figueiredo et al., 2019; Ayari et al., 2020; Cho et al., 2020). Two combinations (Thy plus bioAgNP, Table 4, and Car plus Thy, Table 5) reduced significantly the MIC values for 90% of the tested strains, showing an additive antibacterial interaction. None of the four combinations showed an antagonistic antimicrobial interaction for all the tested strains.

This study showed, for the first time, the potent antibacterial activity of *F. oxysporum*-bioAgNP combined to Car and Thy, and that combinations of the oregano derivatives (OEO, Car, and Thy) and the bioAgNP have a potent antibacterial activity against carbapenem-resistant strains. The combination composed by Thy and bioAgNP not only reduced required dose of each compound to inhibit bacterial growth; also showed antibacterial action in shorter time compared to individual bioAgNP, decreasing time required for 5 log reduction from 4 h to 10 min to KPC-producing *K. pneumoniae*.

The reference and multidrug-resistant strains showed similar sensitivity to the oregano derivatives (OEO, Car, and Thy) and bioAgNP, individually and in combination (Table 1). Resistance mechanisms of conventional antimicrobials did not make bacteria also tolerant to terpenoids or bioAgNP. Therefore, the mechanism of action of these alternative antimicrobials may not be related to these resistance mechanisms. It is also the first time that an initial characterization of the mechanism of action of Thy plus bioAgNP (produced by *F. oxysporum*) against *E. coli* was performed. This combination was chosen among others, because we observed that Thy is the oregano derivative with less organoleptic effect, and that this combination showed additive effect against most of the tested strains.

Minimum inhibitory concentration represents one particular degree of antibacterial effect, which produces dramatic changes in bacteria. Subinhibitory concentration also produces effects on bacterial growth, cell morphology, ultrastructure, and virulence (Zhanel et al., 1992; Vasilchenko and Rogozhin, 2019). Our short time-kill assay showed the ideal concentrations of antibacterials to be used in the initial study on their mechanisms of action. Concentrations that did not cause reduction in bacterial inoculum were chosen, so the identified bacterial alterations may be due to the action of antimicrobials, not due to cell death process, which represents indirect bacterial changes.

The SEM analysis (Figure 6) showed that all the tested antimicrobials (OEO, Car, Thy, bioAgNP, and combination of Thy and bioAgNP) resulted in highly deformed cells, causing physical damage and considerable morphological changes (surface blebbing) in *E. coli*, as confirmed by the other assays (ATP, membrane leakage of biomolecules, and oxidative stress tests). The surface protrusions indicate disruption of the cellular wall and cytoplasmic membrane, and the cytoplasmic material being released was in agreement with other studies (Kim et al., 2011; Scandorieiro et al., 2016; Figueiredo et al., 2019). Thy, bioAgNP, and their combination caused oxidative stress that resulted in high MDA production; lipid peroxidation reduced membrane fluidity, which altered the properties of this structure and may disrupt membrane-bound proteins (Cabiscol et al., 2000). This suggests that Thy, bioAgNP, and their combination may damage the cell membrane directly or indirectly by oxidative stress. OEO and Car acted as antioxidants (ROS assay), which suggests that both damage the cell membrane directly.

In the SEM micrographs, cells with no typical size are observed in all the treatments. Increase in cell size is evident in two samples, the OEO-treated sample and Thy plus bioAgNP- treated sample (cells looked turgid compared to the untreated control); such results probably are related to the release of internal cell material that may affect bacterial osmoregulatory capacity (Hartmann et al., 2010), and to cell wall damage that probably resulted in lost of ability to limit bacterial volume.

The MET study (Figure 7) indicated that bioAgNP treatment caused disruption of the cellular wall and cytoplasmic membrane, and decrease in electron density compared to the untreated control, corroborating the SEM and other assay results, which confirmed that the bioAgNP induce bacteria to release cellular material. The cytoplasmic membrane is one of the most active structures of a bacterium, being responsible for most of its cellular functions. BioAgNP can sustainably release Ag^+^ that binds to sulfhydryl groups in enzymes and proteins (Qing et al., 2018). Some studies suggest bioAgNP antibacterial action involves mainly Ag ion, and others researchers highlight the importance of intrinsic effects of nanoparticle (Despax et al., 2011; Yin et al., 2011; Levard et al., 2012; Xiu et al., 2012; Kędziora et al., 2018; Qing et al., 2018). Although their mechanism of action is not fully understood, it is known which bacterial structures are affected by bioAgNP. Since bioAgNP cause damage to bacterial membranes, countless negative consequences may happen to cells such as dissipation of proton motive force, collapse of membrane potential, depletion of intracellular ATP level, damage to respiratory chain, and destabilization of the outer membrane of the cell wall (Lok et al., 2006; Li et al., 2010; Kim et al., 2011; Dakal et al., 2016; Qing et al., 2018). Huq (2020) reported that green-synthetized silver nanoparticles cause morphological and ultrastructural changes in *S. aureus* and *P. aeruginosa*; SEM analysis showed irregularly wrinkled, damaged, deformed and cracked outer surfaces. Feng et al. (2000), Li et al. (2010), and Ninganagouda et al. (2014) showed by electron microscopy that nanosilvers cause damage to the bacterial cell membrane and wall, leading to disruption of such structures and causing release of the cytoplasmic material.

If ROS increase intensely, it can lead to oxidative stress. Oxidative stress can result in damage to cell lipids, proteins, and DNA. Example of ROS include superoxide (O^2–^), hydroxyl radical (OH^–^), hydrogen peroxide (H_2_O_2_), among others (Woolley et al., 2013; Flores-López et al., 2019). In this study, we used probe fluorescein isothiocyanate (FITC), which reacts with nucleophiles such as amine, sulfhydryl groups, and the phenolate ion of tyrosine in proteins; the oxidation-reduction process occurs between ROS and a reduced probe, which fluoresces upon oxidation (Hermanson, 2013; Woolley et al., 2013). Lipid peroxidation is also a marker for oxidative stress; it indicates the lipid degradation that happens as result of oxidative damage. Polyunsaturated lipids are susceptible to oxidative attack by ROS, resulting in end products such as malondialdehyde (MDA) (Cabiscol et al., 2000; Tsikas, 2017). In this study, lipid peroxidation was detected by the reaction between thiobarbituric acid (TBA) and MDA.

In this study, healthy untreated bacterial cells produced ROS as natural bioproducts of aerobic respiration (Muras et al., 2019). Thy and bioAgNP alone significantly caused ROS production in *E. coli* compared to the untreated control, in line with other studies (Kim et al., 2011; Li et al., 2014; Ninganagouda et al., 2014; Shen et al., 2016; Yan et al., 2018; Al-Kandari et al., 2019); the combination of Thy and bioAgNP also increased ROS production by *E. coli*. In addition to protein oxidation, lipid oxidation also occurred. The lipid peroxidation assay showed that Thy, bioAgNP and their combination caused oxidative stress, which was in line with the ROS assay, since the treated bacterial samples presented significantly higher levels of MDA than the untreated control. Other studies also reported that Thy and AgNP stimulate MDA production (Gao et al., 2016; Thombre et al., 2016; Qing et al., 2018; Quinteros et al., 2018). The oxidative stress data are shown in Figure 3.

In this study, exactly at the beginning of treatment with Thy (0 h), *E. coli* produced significantly higher amount of ROS than the untreated control (Figure 3A). Although the antioxidant activity of Thy has been reported on animal cells (Chauhan and Kang, 2014; Coccimiglio et al., 2016; Nagoor Meeran et al., 2017), some studies have shown that its antimicrobial activity involves ROS production (Shen et al., 2016; Al-Kandari et al., 2019). Yuan et al. (2018) showed that genes related to oxidative stress defense were upregulated in Thy-treated *E. coli* O157:H7. It suggests that Thy might impose oxidative stress on bacteria cells, since their biological aspects of oxidative stress differ from that of animals. Our time-kill assay (Figure 2) and ROS assay (Figure 3A) show that Thy acted against bacterial populations immediately at the beginning of treatment; in this stage, bacteria were not growing, and this condition exposed cells permanently to all the produced amount of ROS; rapid cell division that happens in log phase is a character that reduces damages caused by oxidative stress, since ROS amount is shared among cells (Sigler et al., 1999).

Our data showed that bioAgNP individually or in combination with Thy increased ROS production later (after 1 h of treatment) (Figure 3A). Liao et al. (2019) reported excessive ROS production in AgNP-treated *P. aeruginosa*, and showed that antioxidants (reduced glutathione and ascorbic acid) partially antagonized AgNP antibacterial action. Same researchers showed that AgNP destroyed REDOX homeostasis in a Gram-negative bacterium, causing alteration in gene expression and activity of redox-relevant enzymes (superoxide dismutase, catalase, and peroxidase). Ninganagouda et al. (2014) studied biogenically synthesized AgNP; their results showed that nanoparticles induced ROS production in *E. coli*, and that ascorbic acid reacted as a scavenger hindering excessive ROS-production. Quinteros et al. (2018) reported that AgNP generated oxidative stress in *E. coli* and *S. Aureus* and mediated by increase of ROS, which caused high levels of oxidized proteins and lipids, DNA fragmentation, and modification in membrane potential.

Data with regard to ROS (Figure 3A) are in line with time-kill (Figure 2) that showed the immediate antibacterial action of Thy (10 s) and later action of the bioAgNP (30 min) and their combination (10 min). ROS amount was higher in the Thy-treated sample than the bioAgNP- or combination-treated samples, suggesting that ROS production contributes slightly more to the antibacterial action of Thy than bioAgNP individually or their combination.

*O. vulgare* (oregano) essential oil and Car presented antioxidant activity (Figure 3A), reducing significantly ROS production compared to the control, in agreement to the literature (Rodriguez-Garcia et al., 2015; Gutiérrez-Grijalva et al., 2018; Sharifi-Rad et al., 2018; Hać-Szymańczuk et al., 2019). It has been reported that oregano antioxidant effect is due to flavonoids and phenolic acids such as rosmarinic acid, (-)-epicatechin, chicoric, caffeic acid, eriodictyol, and naringenin (Gutiérrez-Grijalva et al., 2018). Hać-Szymańczuk et al. (2019) reported that addition of OEO helped in prolonging the storage stability of chickens for 9 months (vacuum-packed and stored at frozen temperature) by limiting lipid peroxidation. Car improves the activity of enzymatic antioxidants (superoxide dismutase, catalase, and glutathione peroxidase in rat plasma for example). Car also preserves the quality of seed oils, inhibiting the formation of oxidative products that cause deterioration and undesirable flavors (Quiroga et al., 2014; Sharifi-Rad et al., 2018).

This study indicates that oxidative stress is not a mechanism involved in the antimicrobial activity of OEO and Car against *E. coli*, but that it is important in the mechanism of action of Thy and bioAgNP, and their combination. Despite the oxidizing action of Thy, our results suggest that Car antioxidant action is prevalent in OEO, perhaps because this oil has high amount of Car and small amount of Thy. However, different extraction methods and variations in oil composition may lead to different results (Leyva-López et al., 2017; Gavahian et al., 2018; Gutiérrez-Grijalva et al., 2018).

**FIGURE 9:**
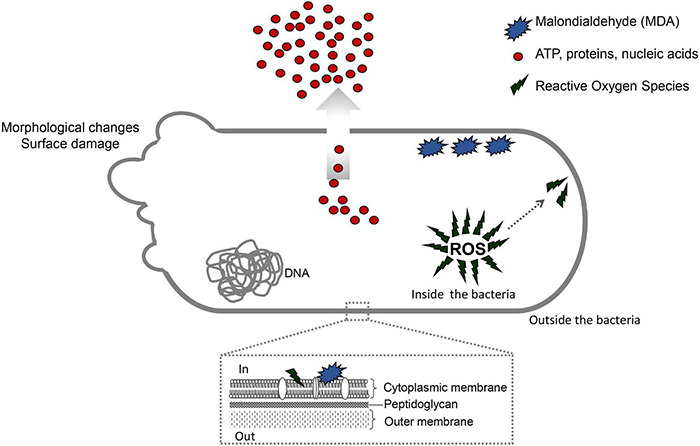
Antibacterial mechanism of action of the bioAgNP and oregano derivatives, both alone and in combination. *Oregano vulgare*-derivatives, especially Thy, disturb the selective permeability of bacterial cell membrane, resulting in high leakage of cellular proteins, DNA, RNA, and ATP. Electron microscopy analysis shows they also cause physical damage in the cytoplasmic membrane and cell wall. The antibacterial mechanism of Thy also involves oxidative stress by enhancing intracellular reactive oxygen species and amount of MDA (marker of lipid peroxidation). The bioAgNP show multiple antibacterial mechanisms: ROS generation, lipid peroxidation, disruption of cytoplasmic membrane and cell wall, and loss of cytoplasmic content. The combination containing Thy and bioAgNP shows strategic antibacterial mechanism. Thy disturbs the selective permeability of the bacterial cell membrane and consequently facilitates access of nanoparticles to the bacterial cytoplasm. Thy plus bioAgNP causes loss of cytoplasmic content, physical damage in the membrane and wall cell, and oxidative stress.

The ATP assay (Figure 4), measurement of cellular released materials (Figure 5), and electron microscopy (Figures 6, 7) analysis suggest that the tested oregano derivatives (OEO, Car, and Thy) and bioAgNP (synthesized with *F. oxysporum* components) affected the cytoplasmic membrane integrity of *E. coli* ATCC 25922 (Figure 9), in agreement to the literature. The same result observed for Thy and bioAgNP individually was detected for the combination of both compounds. In this study, we used a bioluminescence assay based on luciferin-luciferase reaction to measure ATP release from cells. ATP permeates the cell membrane and reacts with external luciferase to produce luminescence with luciferin (Hara, 2009). All the tested antimicrobials caused higher loss of bacterial ATP and/or led to higher cellular content leakage (total proteins, ssDNA, dsDNA, and RNA) compared to the untreated cells, in agreement with other studies (Lok et al., 2006; Caillet et al., 2009; Li et al., 2010; Kim et al., 2011; Nazzaro et al., 2013; Souza et al., 2013; Thombre et al., 2016; Khan et al., 2017; Nowotarska et al., 2017; Qing et al., 2018). Khan et al. (2017) showed that Car altered membrane permeabilization in *E. coli*, since this terpenoid caused release of cellular materials such as DNA and proteins, induced significant reduction in membrane electrical potential, increased crystal violet uptake, and induced structural disruption on cell surfaces detected by SEM and fluorescence microscopy using acridine orange and ethidium bromide. Several studies indicate that OEO, Car, and Thy displayed several effects on bacterial surface structures such as increased membrane permeability, cellular material release, membrane depolarization, physical change in cell surface, inhibition of efflux pumps, inhibition of membrane ATPases, reduction in intracellular ATP levels, inorganic phosphate and other ions, and bacterial lysis (Xu et al., 2008; Nazzaro et al., 2013; Scandorieiro et al., 2016; Khan et al., 2017; Kachur and Suntres, 2020).

We highlight that Thy caused greater release of ATP (Figure 4), in absolute values, compared to other treatments. The oregano-derived compounds (OEO, Car and Thy) caused higher protein, DNA, and RNA loss than other the treatments (which also caused intracellular material lost, Figure 5). The ATP and biomolecule leakage assays suggest that damage to cell membrane contributes more to the antibacterial mechanism of action of essential oils (or terpenoids) than bioAgNP.

In present study, OEO (8.1 mg/ml), Car (4.1 mg/ml), and Thy (0.6 mg/ml) showed low toxicity to RBC, in agreement with other studies, since most of the tested bacteria were inhibited by the oregano derivatives at concentrations below CC_50_ (values in parentheses indicate CC_50_). Our previous study with another OEO batch has shown that this oil was not toxic to human blood cells (Scandorieiro et al., 2016). Cacciatore et al. (2015) reported that Car did not show hemolytic activity at its MIC values.

The bioAgNP produced with the *F. oxysporum* method were not cytotoxic to human RBCs (Figure 8), since their CC_50_ was 121.9 μg/ml and MIC ranged from 15.7 to 31.5 μg/ml. The spectrophotometric analysis indicated that the bioAgNP caused minimal damage to blood cells and very little hemoglobin loss at concentrations, which are efficient against bacteria. Other studies have also shown the minimal toxicity of bioAgNP (Choi et al., 2011; de Lima et al., 2012; Marcato et al., 2013; Scandorieiro et al., 2016). Marcato et al. (2013) reported that bioAgNP (also produced with *F. oxysporum*) showed no toxic effect on fibroblast at their effective concentrations against bacteria. Choi et al. (2011) studied AgNP whose CC_50_ against RBC was 700 μg/ml.

The same bioAgNP studied by our research team showed toxicity against HEp-2 cells at MIC against bacteria (Longhi et al., 2016; Scandorieiro et al., 2016), probably due to their antitumor action (Shi et al., 2016; da Silva et al., 2018; Hembram et al., 2018). Several studies indicate that the toxicity of these metal nanoparticles is dependent on several factors such as size, morphology and capping agents (Hanan et al., 2018; Hembram et al., 2018). However, biogenic AgNPs are eco-friendly and less toxic than chemically synthesized nanoparticles, since chemical reagents are not used as reducing or stabilizing agents (de Lima et al., 2012). Our results indicated that the fungal-free solution showed no hemolytic activity (Figure 8) and no antibacterial activity (data not shown), suggesting that bioAgNP biological activity is due to silver nanoparticles and not to fungal traces.

The compounds, in combination, were non-toxic to erythrocytes at MIC values, since they did not cause even 50% of hemolysis (CC_50_ of combinations was not found). All the tested combined concentrations between the oregano compounds and bioAgNP (OEO + bioAgNP, Car + bioAgNP, Thy + bioAgNP, and Car + Thy) did not cause high degree of hemolysis; even additive effect (shown as antibacterial) between compounds did not affect RBCs. Thy alone showed greater hemolytic activity than the other compounds (Figure 8); however, Thy in combination with Car or bioAgNP was not toxic to RBCs at their MIC values (Table 6).

In conclusion, the combination of Thy and bioAgNP showed an additive antibacterial action against multidrug-resistant Gram-positive and negative strains at low doses and had an extremely fast action. Its mechanism of action involves oxidative stress by enhancing intracellular ROS, which consequently caused a significant increase in MDA production (marker for lipid peroxidation). Thy plus bioAgNP also disrupted the *E. coli* cytoplasmic membrane and cell wall (microscopy-proven damage), resulting in release of cellular proteins, DNA, RNA, and ATP (Figure 9). Therefore, Thy, combined with the bioAgNP has a potential to be applied in industries (food package, cosmetic products, formulation of surface cleaners, for example), human and veterinary clinical and hospital settings (wound care supplies, for treating infection in burns, disinfectant products, for example), among others.

## Data Availability Statement

The original contributions presented in the study are included in the article/Supplementary Material, further inquiries can be directed to the corresponding author.

## Author Contributions

SS contributed to the conception and drafting of the study, design and planning of the experiments, carrying out the experiments, data acquisition, analysis, and interpretation, and writing of this article. BR carrying out the MIC and time-kill assays, data acquisition, analysis, and interpretation, and critical review of the article. EN contributed to the designing and planning of the stress oxidative, ATP, and transmission electron microscopy assays. LP contributed to the assistance and guidance in the bioAgNP biosynthesis, mainly with fungal growth, and stock conditions. AO contributed to the assistance and guidance in the electron microscopy assays. ND contributed to the conception of the bioAgNP biosynthesis methodology and nanoparticle characterization. GN contributed to the assistance and guidance in the bioAgNP biosynthesis, data analysis and interpretation, and critical review of the article. RK contributed to the conception and advisor of this study, encouragement to SS to investigate the mechanism of action of the antimicrobials, data analysis and interpretation, critical review of the article, and final approval of the version to be published. All authors contributed to the article and approved the submitted version.

## Conflict of Interest

The authors declare that the research was conducted in the absence of any commercial or financial relationships that could be construed as a potential conflict of interest.

## Publisher’s Note

All claims expressed in this article are solely those of the authors and do not necessarily represent those of their affiliated organizations, or those of the publisher, the editors and the reviewers. Any product that may be evaluated in this article, or claim that may be made by its manufacturer, is not guaranteed or endorsed by the publisher.

## Abbreviations

OEO: *Oregano vulgare* (oregano) essential oil
Car: carvacrol
Thy: thymol
bioAgNP: biogenically synthesized silver nanoparticles.
