# Supplementary material for: Biogenic Silver Nanoparticles Strategically Combined With Origanum vulgare Derivatives: Antibacterial Mechanism of Action and Effect on Multidrug-Resistant Strains
Source: Front Microbiol. 2022 May 6;13:842600. doi: 10.3389/fmicb.2022.842600 (PMC9121793; doi:10.3389/fmicb.2022.842600)
Supplement: Supplementary file 1 [file Data_Sheet_1.docx]

Supplementary Material

**Content list of this supplementary file**

**Supplementary Tables:**

**Supplementary Table 1.** Antimicrobial susceptibility of clinical isolates.

**Supplementary Table 2.** Determination of subinhibitory concentrations of oregano derived antimicrobials and bioAgNP individually and in combination for mechanism assays.

**Supplementary Table 3.** Details about prolonged exposition of *Escherichia coli* ATCC 25922 to Thy and AgNP (individually and in combination).

**Supplementary Figures:**

**Supplementary Figure 1.** Size distribution by intensity of bioAgNP.

**Supplementary Figure 2.** Zeta potential distribution of bioAgNP.

# Supplementary Tables

| **Supplementary Table 1.** Antimicrobial susceptibility of clinical isolates. | | | | | | | | | | | | | | | | | | | | | | | | | | |
| --- | --- | --- | --- | --- | --- | --- | --- | --- | --- | --- | --- | --- | --- | --- | --- | --- | --- | --- | --- | --- | --- | --- | --- | --- | --- | --- |
| **Enterobacteriaceae** | **AMP** | **AMC** | | **CFZ** | **FEP** | | **CFO** | **CAZ** | | **CAZ**  **CLA** | **ATM** | | | **ETP** | **GEN** | | **STR** | **TET** | | **CIP** | **NOR** | | **NAL** | **CLO** | **FO** | **NIT** |
| *E. coli* KPC 126^(a)^ | R | R | | R | R | | R | R | | R | R | | | R | R | | R | R | | R | R | | R | R | S | S |
| *K. pneumoniae* KPC 5795^(b)^ | R | R | | R | R | | R | R | | R | R | | | R | S | | S | S | | R | R | | R | S | S | R |
| ***P. aeruginosa*** | **CAZ** | | **CAZ**  **CLA** | | | **FEP** | | | **ATM** | | | | **IPM** | | | **PB** | | | **GEN** | | | **CIP** | | | **NOR** | |
| 3400^(c)^ | S | | S | | | R | | | R | | | | R | | | S | | | R | | | R | | | R | |
| ***A. baumannii*** | **AMS** | | **CAZ** | | | **CAZ**  **CLA** | | | **FEP** | | | **CTX**  **CLA** | | | | **IPM** | | | **GEN** | | | **TET** | | | **CIP** | |
| CR 01^(d)^ | R | | R | | | S | | | R | | | R | | | | R | | | S | | | R | | | R | |

*AMC, Amoxicillin-clavulanate; AMP, Ampicillin; AMS, Ampicillin-sulbactam; ATM, Aztreonam; CAZ CLA, Ceftazidime-clavulanate; CAZ, Ceftazidime; CFO, Cefoxitin; CFZ, Cefazolin; CIP, Ciprofloxacin; CLO, Chloramphenicol; CTX CLA, Cefotaxime-clavulanate; ETP, Ertapenem; FEP, Cefepime; FO, Fosfomycin; GEN, Gentamicin; IPM, Imipenem; NAL, Nalidixic acid; NIT, Nitrofurantoin; NOR, Norfloxacin; PB, Polymyxin B; STR, Streptomycin; TET, Tetracycline.*

*KPC, K. pneumoniae Carbapenemase; CR, Carbapenem-resistant.*

*126, 5795, 3400, and 01 are strain numbers from Laboratory of Basic and Applied Bacteriology - UniversidadeEstadualdeLondrina.*

*R, Resistant; S, Susceptible.*

*(a) isolate from urinary tract infection;(b) isolate from surgical wound swab; (c) isolate from tracheal secretion; (d) isolate from inguinal-rectal swab.*

| **Supplementary Table 2.** Quantification of viable cells (log CFU/mL) of *Escherichia coli* ATCC 25922 at five time points of treatments to determine subinhibitory concentrations of oregano derived antimicrobials and bioAgNP individually and in combination. | | | | | |
| --- | --- | --- | --- | --- | --- |
| **Treatments** | **0.008 h** | **0.5h** | **1h** | **2h** | **3h** |
| **Control** | 8.99 ± 0.01 | 8.95 ± 0.08 | 9.08 ± 0.17 | 9.16 ± 0.09 | 9.08 ± 0.07 |
| **OEO 0.3 mg/mL** | 7.46 ± 0.03 | 6.82 ± 0.17 | 4.75 ± 0.08 | 2.88 ± 0.11 | 2.60 ± 0.11 |
| **OEO 0.15 mg/mL^(a)^** | 9.10 ± 0.03 | 9.03 ± 0.12 | 9.10 ± 0.02 | 9.04 ± 0.04 | 8.95 ± 0.05 |
| **Car 0.31 mg/mL** | 7.08 ± 0.00 | 5.31 ± 0.01 | 4.00 ± 0.00 | 0.00 ± 0.00 | 0.00 ± 0.00 |
| **Car 0.15 mg/mL^(a)^** | 9.03 ± 0.05 | 9.03 ± 0.02 | 8.99 ± 0.07 | 9.03 ± 0.05 | 9.04 ± 0.11 |
| **Thy 0.25 mg/mL** | 3.05 ± 0.05 | 0.00 ± 0.00 | 0.00 ± 0.00 | 0.00 ± 0.00 | 0.00 ± 0.00 |
| **Thy 0.12 mg/mL^(a)^** | 9.00 ± 0.04 | 9.09 ± 0.05 | 9.05 ± 0.05 | 9.04 ± 0.04 | 9.05 ± 0.05 |
| **bioAgNP 15.75 µg/mL^(a)^** | 9.07 ± 0.02 | 9.07 ± 0.14 | 9.04 ± 0.00 | 9.08 ± 0.04 | 9.17 ± 0.08 |
| **Thy 0.06 mg/mL + bioAgNP 7.88 µg/mL** | 8.52 ± 0.14 | 7.37 ± 0.05 | 6.43 ± 0.17 | 4.00 ± 0.01 | 4.00 ± 0.00 |
| ***Thy 0.03 mg/mL + bioAgNP 7.88 µg/mL^(a)^** | 9.03 ± 0.05 | 9.04 ± 0.04 | 8.99 ± 0.05 | 9.05 ± 0.06 | 8.97 ± 0.06 |

*OEO; Oregano essential oil; CAR; Carvacrol; Thy, Thymol; bioAgNP, Biogenically synthesized silver nanoparticles.*

*Values of CFU/mL are the mean ± standard deviation.*

*^(a)^Antimicrobials at subinhibitory concentrations.*

**Supplementary Table 3.** Prolonged exposition of *Escherichia coli* ATCC 25922 to Thy and bioAgNP (individually and in combination) for 25 days. The highest antimicrobial concentrations that do not inhibit bacterial growth are indicated in table.

| **Day** | **Thy** | **bioAgNP** | **Thy + bioAgNP** |
| --- | --- | --- | --- |
| **1** | 0.12 × MIC | 0.12 × MIC | 0.12 × MIC |
| **2** | 0.12 × MIC | 0.12 × MIC | 0.12 × MIC |
| **3** | 0.12 × MIC | 0.12 × MIC | 0.12 × MIC |
| **4** | 0.12 × MIC | 0.12 × MIC | 0.12 × MIC |
| **5** | 0.25 × MIC | 0.25 × MIC | 0.25 × MIC |
| **6** | 0.25 × MIC | 0.25 × MIC | 0.25 × MIC |
| **7** | 0.25 × MIC | 0.25 × MIC | 0.25 × MIC |
| **8** | 0.25 × MIC | 0.25 × MIC | 0.25 × MIC |
| **9** | 0.25 × MIC | 0.5 × MIC | 0.5 × MIC |
| **10** | 0.4 × MIC | 0.5 × MIC | 0.5 × MIC |
| **11** | 0.4 × MIC | 0.5 × MIC | 0.5 × MIC |
| **12** | 0.4 × MIC | MIC | 0.8 × MIC (Thy) + MIC (Ag) |
| **13** | 0.4 × MIC | MIC | 0.8 × MIC (Thy) + MIC (Ag) |
| **14** | 0.4 × MIC | 1.2 × MIC | 0.5 × MIC (Thy) + MIC (Ag) |
| **15** | 0.4 × MIC | 1.4 × MIC | 0.6 × MIC (Thy) + MIC (Ag) |
| **16** | 0.4 × MIC | 1.6 × MIC | 0.6 × MIC (Thy) + MIC (Ag) |
| **17** | 0.4 × MIC | 1.8 × MIC | 0.8 × MIC (Thy) + MIC (Ag) |
| **18** | 0.4 × MIC | 2 × MIC | 0.9 × MIC (Thy) + MIC (Ag) |
| **19** | 0.4 × MIC | 2.2 × MIC | 0.9 × MIC (Thy) + MIC (Ag) |
| **20** | 0.5 × MIC | 2.8 × MIC | MIC |
| **21** | 0.5 × MIC | 3.4 × MIC | 0.9 × MIC (Thy) + MIC (Ag) |
| **22** | 0.6 × MIC | 4.5 × MIC | 0.9 × MIC (Thy) + MIC (Ag) |
| **23** | 0.6 × MIC | 5.5 × MIC | 0.9 × MIC (Thy) + MIC (Ag) |
| **24** | 0.6 × MIC | 11 × MIC | 0.9 × MIC (Thy) + MIC (Ag) |
| **25** | 0.6 × MIC | 11 × MIC | 0.9 × MIC (Thy) + MIC (Ag) |

*Thy, Thymol; AgNP, biologically synthesized silver nanoparticles.*

*MIC, Minimum inhibitory concentration.*

*Thy MIC = 0.25 mg/mL*

*bioAgNP MIC = 15.75 µg/mL*

*MIC of antimicrobials in combination (Thy = 0.12 mg/mL; bioAgNP= 7.88 µg/mL)*

*Bacterial sample that was treated with combination did not develop tolerance to bioAgNP, whose MIC remained 15.75 µg/mL.*

# Supplementary Figures

**
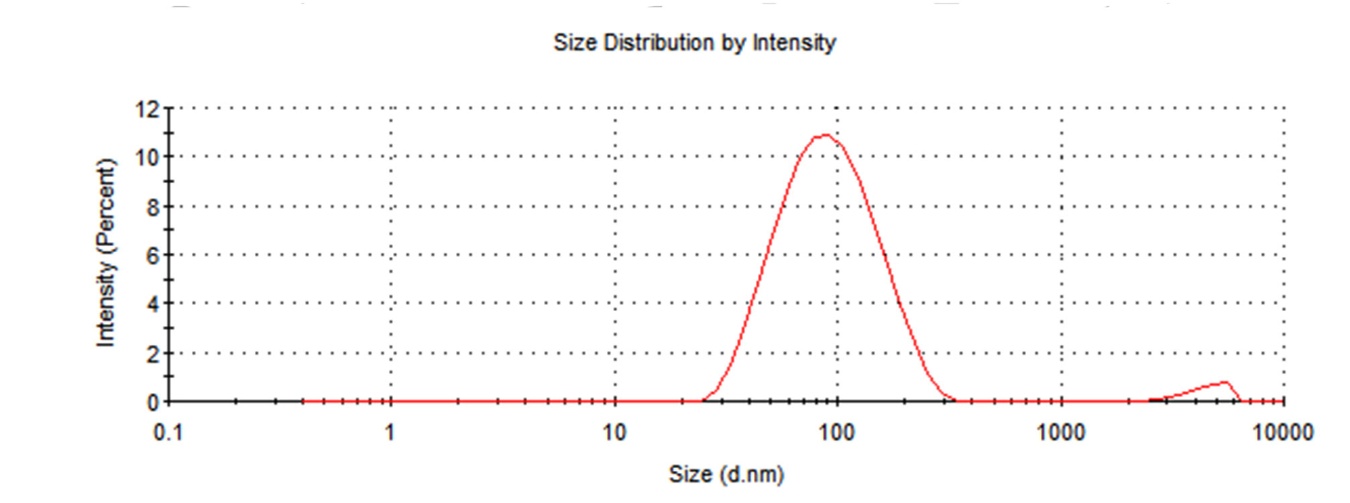
**

**Supplementary Figure 1.** Size distribution by intensity (%) of biologically synthesized silver nanoparticles (bioAgNP). Based on all of the intensities provided by photon correlation spectroscopy, the average diameter of bioAgNP was 73 nm. The polydispersity index (PDI) was 0.357.


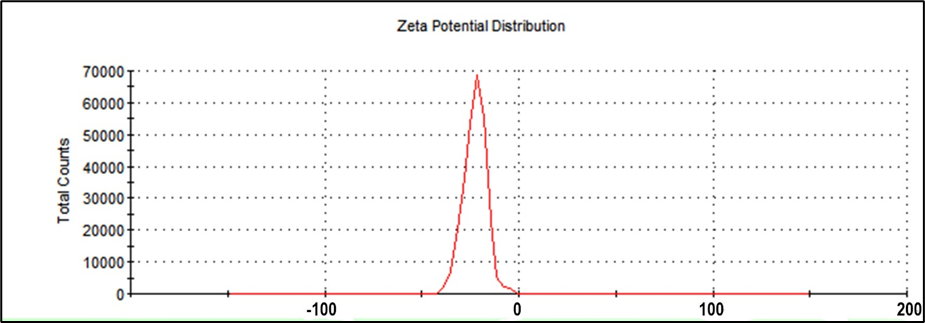


**Supplementary Figure 2.** Zeta potential distribution of biologically synthesized silver nanoparticles (bioAgNP) determined using Zetasizer NanoZS (Malvern). The average zeta potential value of was ‑ 24 mV.
